# Time Constraints Do Not Limit Group Size in Arboreal Guenons but Do Explain Community Size and Distribution Patterns

**DOI:** 10.1007/s10764-018-0048-4

**Published:** 2018-07-11

**Authors:** Amanda H. Korstjens, Julia Lehmann, R. I. M. Dunbar

**Affiliations:** 1 Department of Life and Environmental Sciences, Bournemouth University, Poole, BH14 5BB UK; 2 Department of Life Sciences, University of Roehampton, London, SW15 5PJ UK; 3 Department of Experimental Psychology, University of Oxford, Oxford, OX1 3UD UK

**Keywords:** Biogeography, Cercopithecinae, Group community ecology, Interspecific competition, Intraspecific competition, Living

## Abstract

To understand how species will respond to environmental changes, it is important to know how those changes will affect the ecological stress that animals experience. Time constraints can be used as indicators of ecological stress. Here we test whether time constraints can help us understand group sizes, distribution patterns, and community sizes of forest guenons (*Cercopithecus/Allochrocebus*). Forest guenons typically live in small to medium sized one-male–multifemale groups and often live in communities with multiple forest guenon species. We developed a time-budget model using published data on time budgets, diets, body sizes, climate, and group sizes to predict maximum ecologically tolerable group and community sizes of forest guenons across 202 sub-Saharan African locations. The model correctly predicted presence/absence at 83% of these locations. Feeding-foraging time (an indicator of competition) limited group sizes, while resting and moving time constraints shaped guenon biogeography. Predicted group sizes were greater than observed group sizes but comparable to community sizes, suggesting community sizes are set by competition among guenon individuals irrespective of species. We conclude that time constraints and intraspecific competition are unlikely to be the main determinants of relatively small group sizes in forest guenons. Body mass was negatively correlated with moving time, which may give larger bodied species an advantage over smaller bodied species under future conditions when greater fragmentation of forests is likely to lead to increased moving time. Resting time heavily depended on leaf consumption and is likely to increase under future climatic conditions when leaf quality is expected to decrease.

**Electronic supplementary material:**

The online version of this article (10.1007/s10764-018-0048-4) contains supplementary material, which is available to authorized users.

## Introduction

Conventional ecological theory assumes that closely related species should diverge ecologically or segregate spatially to avoid competition (Ricklefs and Miller 1999). Interspecific competition would seem especially important where species are closely related to each other. However, closely related species regularly share habitats (phylogenetic clumping) because they share the required biological traits that increase their success in particular habitats (Ricklefs 2015) or because their distributions are shaped by dispersal distance since divergence (i.e., neutral theory: Rosindell *et al.* 2011). Phylogenetic clumping is common in African primate communities (Beaudrot and Marshall 2011; Kamilar *et al.* 2015), although environmental variables are also important (Fleagle *et al.* 1999; Kamilar 2009; Reed and Fleagle 1995). Therefore, to understand the drivers behind species’ distributions, we need to understand how biogeography is shaped by both environmental factors and interspecific competition, especially for species that live in communities made up of several closely related species.

We can understand the outcome of the ecological and competitive constraints acting on an individual by studying time budgeting decisions: an individual could survive in any location if it can meet its energetic requirements within the species-relevant time frame and avoid predation (Southgate 1991). Species-relevant time frames depend on physiological cycles, which can range from one day for small animals to several days for larger animals (Peters 1983). Therefore, time constraints can be used as indicators of ecological stress in animals, either directly or indirectly through their effects on energy budgets (Dunbar *et al.* 2009). Time budgets consist mostly of feeding-foraging time, moving time, resting time, and social time (in social animals) (Pollard and Blumstein 2008). The number of competitors in foraging groups is expected to influence moving and feeding-foraging time. Moving time is expected to increase with group size, as larger groups deplete food sources faster, forcing individuals to travel to new food patches (Chapman and Chapman 2000; Janson and van Schaik 1988). Feeding-foraging time is expected to increase with group size, as competition forces individuals to spend more time foraging (i.e., searching) for food that is depleting faster or to spend more time feeding because individuals switch to lower quality foods to make up for shortfalls in high-quality foods (Janson and van Schaik 1988; Pazol and Cords 2005). For most diurnal primates, social group size is the best indicator of foraging group size because of the cohesive nature of the social groups. In multispecies associations of closely related species, the presence of individuals from other species is also expected to result in competition. The best indicator of foraging group size in this case is community size (defined here as the total number of individuals of all closely related species in the area). Still, the effect of community size on feeding-foraging and moving time would be expected to be less strong than that of group size because of niche separation (Barabás *et al.* 2016). Climatic conditions are expected to have a mediating effect on these relationships, as they determine the quality of the environment and thus the amount of resources available per individual (Grove 2012; Marshall *et al.* 2012). The use of time budgets to index ecological constraints on animals has provided insights into intraspecific competition and ecological constraints on individual taxa that differ widely in social system, diet, and distribution patterns (spider monkeys, *Ateles* spp*.*: Korstjens *et al.* 2006; great apes, *Pan* and *Gorilla*: Lehmann *et al.* 2008; various primate species Pollard and Blumstein 2008; red, *Piliocolobus*, and black-and-white, *Colobus*, colobus: Korstjens and Dunbar 2007; vervets, *Chlorocebus*: Willems and Hill 2009; baboons, *Papio*: Bettridge *et al.* 2010; orang-utans, *Pongo*: Carne *et al.* 2012; barbary macaques, *Macaca sylvanus*: Ménard *et al.* 2013; feral goats, *Capra hircus*: Dunbar and Shi 2013; reviewed in Dunbar *et al.* 2009 and discussed in Marshall *et al.* 2012). The effects of interspecific competition on time budgets are less well investigated.

The forest guenon group (genera *Cercopithecus* and *Allochrocebus*) is the ideal primate taxon in which to study the interspecific and environmental determinants of species’ distributions. Forest guenons are among the most diverse African primate taxa, with more than 20 species that are ecologically and phylogenetically very similar, yet they often occur in multispecies communities of up to 7 species (Kingdon and Groves 2013). Multispecies forest guenon associations are common in these communities (often intermingling for at least an hour and as much as full days: Cords 1990; Gautier-Hion and Tutin 1988). Historical changes in forest cover and dispersal ability rather than environmental filtering appear to be the main drivers behind the distributions of individual forest guenon species and speciation patterns (Grubb 1982; Kamilar *et al.* 2009; Tosi *et al.* 2005). Current climatic variables are good predictors of the taxon’s overall distribution (Korstjens 2018). Interspecific competition among forest guenon species in multispecies communities leads to variations among species in strata use and diet (Buzzard 2006; Cords 1986; Eckardt and Zuberbühler 2004; Gautier-Hion 1980; Lambert 2002). However, we do not know how interspecific competition among forest guenon species limits group sizes and continent-wide distributions.

We developed a time-budget model for forest guenons to study the processes that drive guenon–habitat relationships and to identify whether the outcomes of competition among conspecific and allospecific guenons constrain conspecific group sizes. Our time-budget model uses the observed relationships between behavior (time budgets), diet, group size, and climate variables to predict maximum ecologically tolerable group sizes for forest guenons across Africa. We use the insights gained from this modeling approach to discuss how forest guenons may respond to changing climatic conditions.

## Methods

### The Study Species

African guenons, Cercopithecini, are an unusually speciose taxon as a result of a relatively recent radiation (about 5 million years ago: Tosi *et al.* 2005) most likely due to a refugia effect (Tosi 2008). Our analyses focus on the “forest guenons,” characterized by the genus *Cercopithecus* (containing seven species clusters totaling 20 species: Butynski *et al.* 2013). We include the species *Cercopithecus/Allochrocebus lhoesti*, *preussi*, and *solatus* (Kingdon and Groves 2013) in our analyses because recent analyses show they can be considered very closely related or of the same genus (Guschanski *et al.* 2013). Forest guenons are found in forests and woodlands throughout sub-Saharan Africa. They are small to medium-sized, mostly arboreal, diurnal primates that typically occur in groups of 3–17 adult females, 1 male, and their offspring (Glenn and Cords 2002). These species survive on a diet of fruits, complemented by insects or leaves (Electronic Supplementary Material [ESM] Table SI).

### Forest Guenon Behavioral Ecological Data

We collated, from the literature, information on forest guenon group sizes (i.e., the number of individuals of the same species that typically form a cohesive social unit), species richness (= the number of forest guenon species at a site), community size (see later), species time budgets, dietary information (= percentage of fruit/leaf/fauna in the diet), species-specific body mass (in kg; see below), and site-specific climatic data (details below). We did an extensive review of the literature in Web of Science, Google Scholar, and Scopus using the key words guenon, *Cercopithecus*, or *Allochrocebus*, in combination with any of the following terms: activity budget, time budget, diet, group size, density, census, and distribution. We included a study on dietary and time-budget data only if it lasted >10 mo and used rigorous standardized scan sampling or focal animal sampling procedures on multiple individuals; we used group size data from density transects only when observers noted high reliability of the group size of multiple groups or when researchers had studied individual groups in detail. To obtain a good estimate of community size (“estimated community size”) at each site, we calculated the mean group size of each species based on the published literature (ESM Table SII). We then used the cumulative mean group sizes of all species present at a site to estimate community size at that site. A more precise value for community size is not possible, as it would require reliable data on group sizes and home-range sizes of each species at each site, or reliable population density data for all species at all sites. We had group size information for all species present at only 12 sites, and 4 of these were sites with only 1 species. A total of 29 sites had group size information for at least 1 species. We chose to be consistent in our estimate of community size instead of providing observed community sizes only for those sites that are most intensively studied and are not necessarily a representative subset of sites.

We investigated the effect of competition by assessing whether feeding-foraging time and moving time increased with group size, species richness, and/or community size. We used group size as an indicator of the potential for intraspecific competition and community size as an indicator for interspecific competition. We define community size here as the total number of forest guenon individuals within an individual’s range irrespective of species.

In the dataset, we combined foraging time (actively searching for food items such as insects) with feeding time (consumption of food items) because these activities were not consistently separated in all studies. We defined moving time as traveling between feeding or resting locations. Fifteen studies at nine sites and of six species provided data on diet, group size, and time budgets (ESM Table SIII). Dietary data were available for 28 populations from 15 sites, covering 11 species, and group size data for 76 populations from 38 sites, covering 20 species (ESM Tables SI and SII). We used the mean value for variables for which data from several groups of the same species from the same population during the same time period were available.

Because body mass varies between species and can affect group size, time budgets, and diet, we included it as a potential predictor variable for time budgets and diet in the form of the mean of the species-typical values (Butynski *et al.* 2013; Napier 1985; Smith and Jungers 1997) for adult male (BM_am_) and female (BM_af_) body mass (BM_afam) in kg (BM_afam = (BM_af_ + BM_am_)/2). We chose to use the mean of a male and a female body mass because time-budget data are typically collected on both males and females and because the species show greater variation in male than in female body mass. We consider this variation in male body mass an important species-typical biological trait that should be represented in our study.

For all sites used in the analyses, we determined site-specific climatic conditions. Wherever possible we used the researchers’ own climate descriptions; otherwise, we used climatic data from other authors for the same site. We preferred to use site-specific data collected by the researchers because forested areas can have slightly different climatic values from the mean for the local area owing to site-specific conditions, and this provides a closer temporal match between behavioral data and climate data. We derived any missing data from global datasets of monthly and annual temperature and rainfall, and moisture indices in grids of 0.5° latitude by longitude, based on a combination of the Global Historical Climatology Network and weather station records from 1950–1999 inclusive (Legates and Willmott 1990; Willmott and Feddema 1992; Willmott and Matsuura 2001; http://climate.geog.udel.edu/~climate/html_pages/download.html; GHCN version 2). We used the following climate variables (Dunbar *et al.* 2009): mean annual rainfall in mm (P_ann), mean annual mean temperature in °C (T_ann), variation between calendar months in mean monthly temperature (measured as the standard deviation across the 12 mo, T_mo_sd) and in mean monthly rainfall (measured as Shannon’s diversity index across the 12 calendar months, P_mo_sh), the duration of the growing season as determined by the plant productivity index (P>2T, the number of months in the year in which rainfall [in mm] was more than twice the mean monthly temperature [in °C]: Le Houérou 1984), mean annual moisture index (Moi_ann_), and mean monthly moisture index (Moi_mo_). We calculated climate conditions at each of the sites used in this study by taking the mean of climate values of those data points in the Willmott and Matsuura (2001) and the Willmott and Feddema (1992) datasets that fell within a radius of 0.5° longitude and latitude to the site.

### Forest Guenon Distribution Data

To understand how time budgets relate to distribution patterns, we collected information on presence and absence of different genera of cercopithecids (i.e., Old World monkeys) across sub-Saharan Africa for sites where at least one cercopithecid species occurred (ESM Table SIV). We used this selection criterion because we did not want to extrapolate predictions for sites that differed considerably in climatic conditions from those that were used to build the model with. We used the United Nations Environmental Programme (UNEP) World Conservation Monitoring Centre (WCMC) database on protected areas (accessed between 2003 and 2006) and the African Protected Areas Assessment Tool (APAAT), accessed in 2013–2014 (Hartley *et al.* 2007) to locate potential sites. The WCMC and APAAT data are now part of the Digital Observatory for Protected Areas (DOPA) (http://dopa.jrc.ec.europa.eu/node/4). We used Google and Google Scholar to locate reports and scientific publications about primate presence at those locations using the site’s name to verify or complete the data available in the WCMC and APAAT database. If this search produced country- or region-wide primate surveys that included further locations, we added those sites to the dataset. We projected locations of sites onto a map of Africa to identify gaps in site distributions and conducted an intensive search on the Internet to fill those gaps, using the keywords country name with census, primates, mammals, *Cercopithecus*, or monkey. We defined species’ presence as presence of the species within the last 50 years (matching the climate dataset time period), meaning that in some cases the populations have since gone extinct. We verified conflicting or incomplete information by searching for additional reliable scientific reports and publications. We are confident that the distribution data used are highly reliable. Our complete dataset contains 429 sites for which guenon presence/absence is known. To maintain statistical independence and maintain equal coverage across areas, we excluded sites that fell within a radius of 1° of longitude and latitude of another site. If forest guenons were absent at one site but present at a nearby site, we used the site where they were present for our analyses, assuming that the chances that guenons have gone extinct at a site are higher than that a site has a locally atypical climate. This means that some well-known locations did not appear in the dataset used (ESM Table SIV) because they were replaced by smaller nearby sites in the selection process. The resulting dataset contained 202 sites across sub-Saharan Africa: forest guenons were present at 128 of these sites. For 9 of these sites we knew that forest guenons were present but could not reliably establish how many species were present; at the remaining 118 sites we knew the number of species present and could calculate an estimated community size.

### The Time-Budget Model

The time-budget model is designed to determine the maximum ecologically tolerable group size a population can maintain as a coherent social group at a particular site (Dunbar *et al.* 2009). It assumes that an animal has to balance its nutritional intake and expenditure while trading off the different time-budget components (feeding, moving, resting, and affiliative social time/allo-grooming) within each 24-h period. The model is based on observed relationships among climate, group size, diet, and activity budget variables to determine how much time an animal ought to invest in each time-budget component at a specific location (i.e., subject to a specific local climatic regime) as group size increases. Although the number of daylight hours increases with greater distance from the equator, this has a minimal effect on the time-budget model because we use percentage of time obtained from observations collected during winter and summer seasons. Previous time-budget models for *Papio* showed that the effect of latitude was minimal (Dunbar 1992).

Time-budget models are as reliable as niche envelope models, which predict presence and absence based on current distribution patterns and climatic variables directly. The two approaches lead to similar conclusions about the relationship between climatic variables and distribution patterns despite typically small sample sizes for time-budget data compared to large samples for presence/absence used for niche models (Korstjens and Dunbar 2007; Willems and Hill 2009). A niche envelope model for forest guenons based on presence and absence of guenons at 327 sites across Africa had an overall fit of 82%, with a fit of 80% and 84% for predicting absence and presence of guenons respectively (AUC = 0.87, κ = 0.64; Korstjens 2018). The main advantage of the time-budget models over niche envelope models is that time-budget models give an idea about the underlying mechanisms behind species–environment relationships, rather than just extrapolating from presence/absence.

We predicted the maximum ecologically tolerable group size at each location (as the output variable of the modeling process) by determining the group size at which the sum of the individual time-budget variables reaches 100%. Thus, the model predicts not only whether a taxon can be present at a site, but also how well it will do at that site: the ratio between observed group size and predicted group size indexes the ecological stress the animals are under (Dunbar *et al.* 2009). If predicted maximum ecologically tolerable group sizes closely resemble observed mean group sizes then the animals live at maximum group sizes at the expense of suffering greater intragroup competition; if predicted group sizes are much larger than observed group sizes, the animals are split into smaller groups than set by intragroup competition. In all cases, predicted group sizes should be larger than observed mean group sizes because predicted group sizes should identify the group size at which groups have to fission into smaller units, each of which must exceed a minimum viable group size. The need to aggregate to reduce predation risk is expected to set minimum viable group size in diurnal primates but the exact value for this group size is difficult to determine and differs depending on predation pressures (Dunbar *et al.* 2009). Currently we have no tested way of determining that minimum value in forest guenons.

### Building the Model

To create a time-budget model, we first identified the climatic and socioecological parameters that determine the central time-budget components of feeding, moving, resting, and social time (Dunbar *et al.* 2009). We based equations for time spent feeding and moving directly on observed relationships with climatic variables, diet, group size, and adult body mass in forest guenons (ESM Table SIII). We viewed resting time as consisting of “enforced resting time” needed for thermoregulation, sheltering, or digestion and “uncommitted time” that can be converted into more urgent activities when required (Korstjens *et al.* 2010). A comparative study of 83 primate species showed that enforced resting time was best predicted from the percentage of leaves in the diet, mean annual temperature, and temperature variation (Korstjens *et al.* 2010). We used this global equation to calculate minimum required resting time. Using enforced resting instead of total observed values of resting time allows us to identify whether individuals are using up all the available spare time to maintain maximum group sizes or occur in smaller groups with more spare resting time. If predicted group sizes are much larger than observed group sizes, this could be the result of observed resting time values being greater than forced resting time. Social time was calculated using a similar comparative study on average grooming time across 40 primate species (Lehmann *et al.* 2007a, b). When obtaining these global equations, we tested for phylogenetic effects by using the method of phylogenetic generalized least squares (PGLS; Grafen 1989; Garland and Ives 2000). Phylogeny had a small effect on resting time (λ < 0.57) but the equation obtained with phylogenetic corrections was almost identical and had the same model fit (based on AIC values) as the model without phylogenetic corrections (Korstjens *et al.* 2010). The equation for resting time obtained without phylogenetic correction is used here. Phylogeny had no effect on grooming time (λ = 0; Lehmann *et al.* 2007a, b). We calculated percentage of leaves (i.e., vegetative matter) in the diet using an equation obtained from forest guenon diets (ESM Table SI).

We calculated for each location the maximum ecologically tolerable group size at which the sum of the time-budget components reaches 100% under site-specific climatic conditions. First we calculated the baseline time budget at the site (TB_base) as determined by those aspects of our time-budget components that are driven only by climatic conditions. Then, we calculated the group size at each location by subtracting TB_base from 100 and dividing the remaining time by the slope parameters from the equations (in our case the feeding and grooming equation) and rounding down to the nearest whole number. Where predicted group size falls below a chosen cut-off point (see below), a species is predicted to be absent. Resting, feeding, and moving time equation outcomes were all above 3 and well below 99% (the values we set in previous models) except for one case in which resting time was 0.04%. In line with previous models, we reset resting time in that location to 3% to reflect that any animal will have a need to rest at least some time.

### Data Analyses

We used linear mixed model analyses (following Winter 2013), run in R3.3.2 (R Core Team 2016), to derive predictive equations for feeding and moving time and percentage of leaves in the diet, with site and species as random factors and climatic variables, group size, and body weight as independent fixed factors. We tested all variables for normality using skewness and kurtosis (in SPSS 21.0.0.0): only T_ann and the number of guenon species present at a site differed significantly from normality, so these two variables were log_10_ transformed; both were then normally distributed. We used the MuMIn package for R (Bartoń 2016) to select the strongest predictive models with the least number of predictor variables based on AIC and AICc values. Because we are interested in finding equations that are informative and good predictors of primate behavior we selected the most appropriate model from those top models based on whether it was a better fit to the time-budget data than a null model and on whether it made ecological sense (ESM Table SV). We used an ANOVA to test whether the selected linear mixed model performed significantly better than the null model (which included the random factors plus the constant 1; Winter 2013). We used the best-fit model for each time-budget variable (or diet) to provide the regression equation for the time-budget model. For each resulting regression equation, we reviewed partial plots for the relevant variables to check that the equation was not the result of an outlier or an artefact of small sample sizes. Q–Q plots were reviewed to see whether the residuals showed a normal and homogeneous distribution. Because of the limited size of our data sample, we included no more than two predictor variables in an equation. The resting time equation has three predictor variables because it was based on a larger sample size.

### Testing the Model

We validated the model in three ways. First, we assessed the model’s ability to predict forest guenon distribution patterns by analyzing the model’s accuracy in correctly predicting presence/absence of guenons for each of the 202 forest sites. To do so, we calculated predicted group sizes at each site using a generic body mass of 4.4 kg (representing the mean value of observed BM_afam values in our dataset). We determined the accuracy of the model in predicting presence or absence in a binary way by using Cohen’s κ (Cohen 1968; Liu *et al.* 2011), calculated using SPSS 23.0. Predicted group sizes represent our modeled suitability value for each site; i.e., a large predicted group size means presence of guenons is likely and the greater predicted group size is, the more individuals may be present. We used the predicted group size value for which κ reaches its highest value as the minimum suitability value for distinguishing between sites that the model predicts to be suitable vs. unsuitable for forest guenons (Liu *et al.* 2011). Second, we validated the model by comparing predicted to observed group sizes. Because the model is meant to predict the maximum ecologically tolerable group size, predicted group sizes should be higher or equal to observed mean group sizes. Third, we tested the robustness of the model to small changes and sensitivity to large changes in slope parameters by comparing the outcomes of modified models to those of the original model. There are many alternative methods for testing robustness and sensitivity (Pannell 1997) but there is no consensus on the best method. The method we used is intuitively easy to understand and adds to the information obtained from the regression analyses without being dependent on error estimates from those analyses. It is used in individual based modeling (Railsback and Grimm 2012), although researchers often only look for sensitivity (the moderate changes, e.g., Muko *et al.* 2014). Each modified model had one slope parameter changed by plus or minus 10% or 50% for the equations of feeding, leaf-matter, and moving time. This showed whether the relationships we found were robust to slight variation in the equations (10%) but sensitive to large variations (50%) (Dunbar 1984; Dunbar *et al.* 2009; Lehmann *et al.* 2007b, 2008).

Because observed group and community size were not normally distributed, we used nonparametric tests for comparing observed to predicted group sizes (using SPSS 23.0). We used Mann–Whitney *U* tests to compare predicted group sizes or modeled time-budget values of sites where guenons were present and absent. We used Wilcoxon matched pairs tests to compare observed against predicted group sizes and Spearman rank correlation to investigate the relationship among predicted group size, modeled time-budget components, estimated community size, species richness, and observed group size. For the robustness tests, we used the χ^2^ goodness of fit test to compare the predicted presence/absence of the amended model against the outcome of the standard model (as expected values; we adjusted α for multiple tests, *N* = 24, using the Bonferroni method with the Holm’s adjustment (Holm 1979; ESM Table SVI).

#### Data Availability

All data will be available open access via the Bournemouth University’s online research data repository BORDaR (http://bordar.bournemouth.ac.uk/16) and are available in the supplementary materials.

## Results

### Time-Budget and Group Size Determinants

**Fig. 1:**
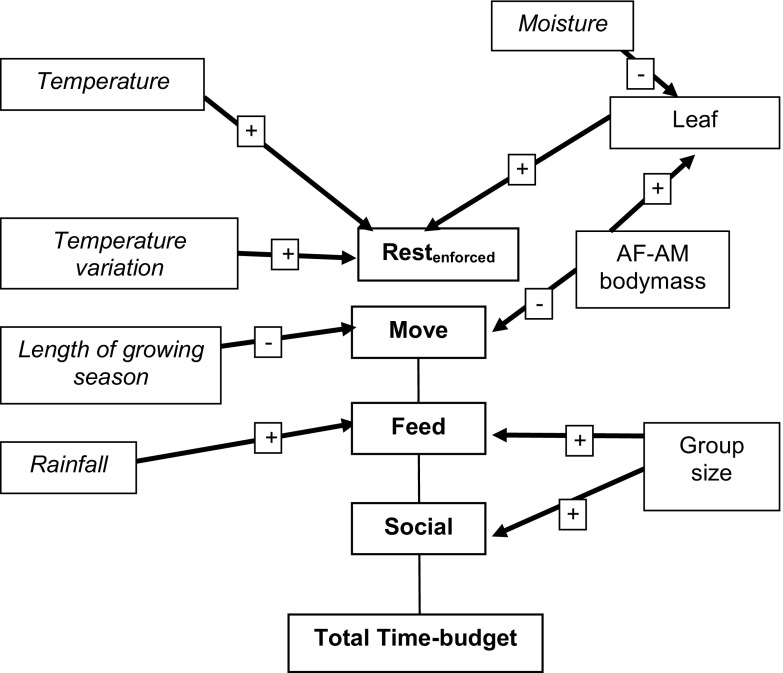
Flow chart for a time-budget model for forest guenons showing how climatic variables, group size, diet, and body mass influence time-budget components. AF = adult female; AM = adult male; length of growing season = number of months in which rainfall (in mm) was greater than twice the mean temperature in that month (°C).

Group size had a significant positive influence on feeding-foraging time but was not required as a predictor to explain moving time (Fig. 1; Table I). All best-fit regression models for moving time that were significantly better than the null model included the mean male–female body mass (details in ESM Table SV). Body mass was an important variable in explaining moving and resting time (through its effect on percentage of leaf in the diet). Species richness and community size (which unfortunately showed limited variation within the time-budget dataset) were not selected as significant contributors to the variation shown in individual time budgets.

**Table I:** Regression equations that best explained the variance in time-budget components in forest guenons with the results of ANOVAs comparing the mixed model (with site and species as random variables) presented against a null model with a constant (=1) and the random variables (see ESM Table SV for further details)

	Equation	df	χ^2^	*P*
Leaf	–2.353 + 5.740 * (mean body mass) – 36.911 * (monthly moisture)	4, 6	7.99	0.018
Feeding/foraging	12.910 + 0.390 * (group size) + 0.00927* (mean annual rainfall)	4, 6	29.56	<0.001
Moving	67.236 – 1.939 * (length of growing season) – 5.032 * (mean body mass)	4, 6	6.85	0.033
Enforced resting	–23.480 + 1.215 * (mean annual temperature) + 0.259 * leaf + 6.647 * (monthly temperature variation)	N/A		
Grooming	1.55 + 0.23* (group size)	N/A		
Baseline time budget	TB_base = (enforced resting) + moving + (12.910 + 0.00927 * (mean annual rainfall)) + 1.55			
Maximum ecologically tolerable group size	= (100 – (TB_base)) / 0.62 (rounded down to the nearest whole number)			

### The Time-Budget Model Validation

**Fig. 2:**
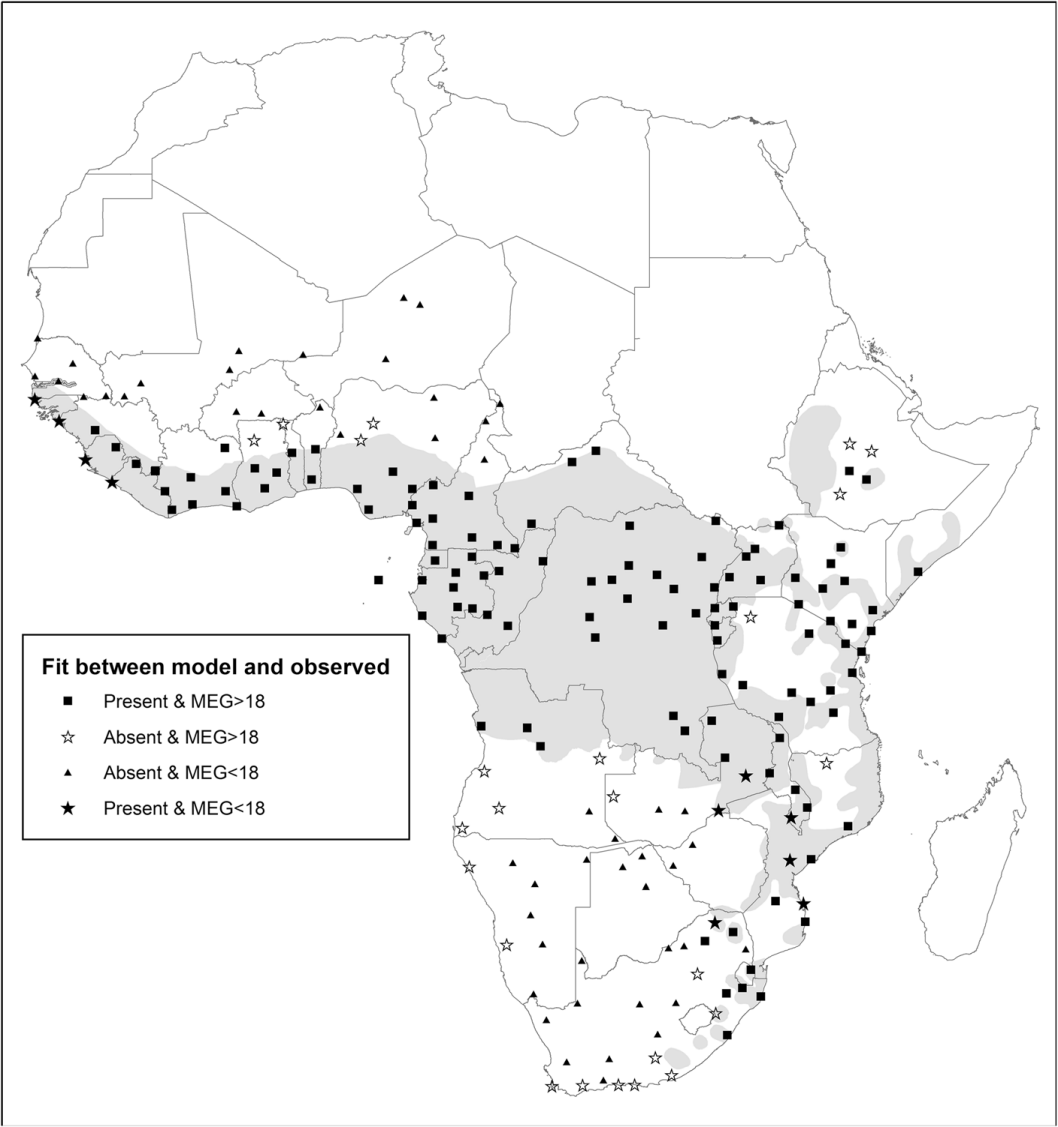
Presence and absence of forest guenons at 202 sub-Saharan African sites and predicted maximum ecologically tolerable group sizes (MEG) for each of the sites (gray regions show approximate current distribution of forest guenons (based on African Mammal Databank [Instituto Ecologia Applicata 1998] range maps for *Cercopithecus* and *Allochrocebus*); some sites where guenons are present fall outside official distribution ranges, suggesting that range maps underrepresent presence.

**Fig. 3:**
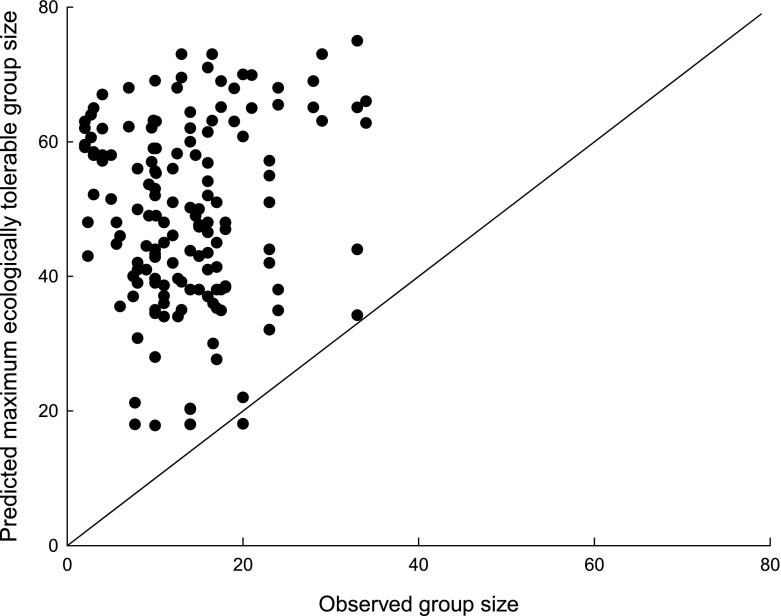
Predicted modeled maximum ecologically tolerable group sizes plotted against observed mean group sizes for 76 forest guenon population across sub-Saharan Africa (the line represents *y* = *x*).

1. Predicting distribution: Using our 202 independent pan-African sites, the time-budget model produced significantly larger group size values at sites where guenons are present (median = 38 individuals; 25th and 75th quartiles: 24.25 and 48.75, *N* = 128) than at sites where they are absent (median = 12.5; 25th and 75th quartiles: 3 and 22, *N* = 74; Mann–Whitney test, *Z* = –8.17, *P* < 0.0001). The value for Cohen’s κ reached its highest value (κ = 0.62) overall when using a minimum predicted group size of 18 individuals as a threshold value for presence (correct absence = 67.57%; correct presence = 92.19%, overall 83.17% correct), showing good agreement with observed distribution patterns (Table II; Fig. 2). The model is expected to predict presence better than absence because absence sites may have recently lost a species or a species may not be able to reach a suitable area because of geographical or human-created barriers.
2. Observed versus predicted group sizes: As required by the logic behind the model, predicted group sizes were greater than observed mean group sizes (one exception; median observed mean group size = 13.00, 25th–75th percentile = 9.38–17.00; predicted group sizes for matching sample of 76 cases: median = 46.95, 25th–75th percentile = 35.62–58.99; Wilcoxon matched pairs test: *Z* = –7.56, *P* < 0.0001) and did not correlate with group size (Spearman’s ρ_groupsize_ = 0.029, *N* = 76, *P* = 0.80; Fig. 3).
3. Robustness tests showed that the model predictions were largely robust against modest errors in the slope parameters in the equations of the model. Of the 12 amended models, in which we adjusted slope parameters (of the diet, moving, and feeding equation) by +10% or –10%, all produced predictions that were not significantly different from those of the base model (α′ = 0.0038, χ^2^ tests presented in ESM Table SV). We recorded the two lowest *P*-values (0.015 and 0.004) in the amended model using a moving equation with the slope parameter for body mass amended by 10%. When we altered slope parameters by +50% or –50%, 10 of the 12 amended models were significantly different from the base model (χ^2^ tests presented in ESM Table SVI, two tests had *P* = 0.048; α′ = 0.0038). The nonsignificantly different amended models were those in which we amended the slope parameter for moisture by 50% in the “leaf” equation. Overall, only a small range of possible values produce the observed results.

**Table II:**
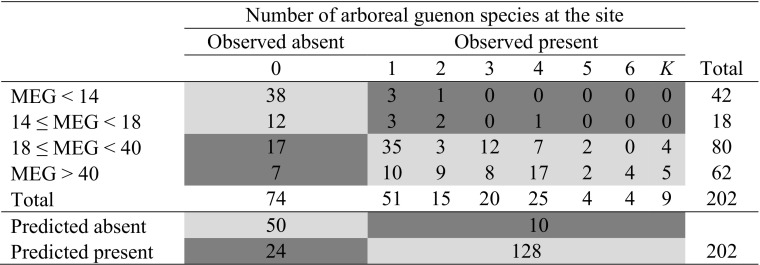
Maximum ecologically tolerable group sizes (MEG) as modeled by a time-budget model against the number of forest guenons present at 202 sub-Saharan African sites

*K* = guenons are present but the total number of species is unknown. The model prediction for presence vs. absence of forest guenons reaches the highest predictive accuracy when MEG < 18 is considered unsuitable for forest guenons (dark gray indicates cases for which modeled values did not match observed values and light gray indicates cases for which modeled presence/absence fitted observed presence/absence).

Thus, the model performed well in these three tests of its validity and showed a good fit with observed forest guenon distributions.

### Processes Driving Species Richness, Group Size, and Biogeography

**Fig. 4:**
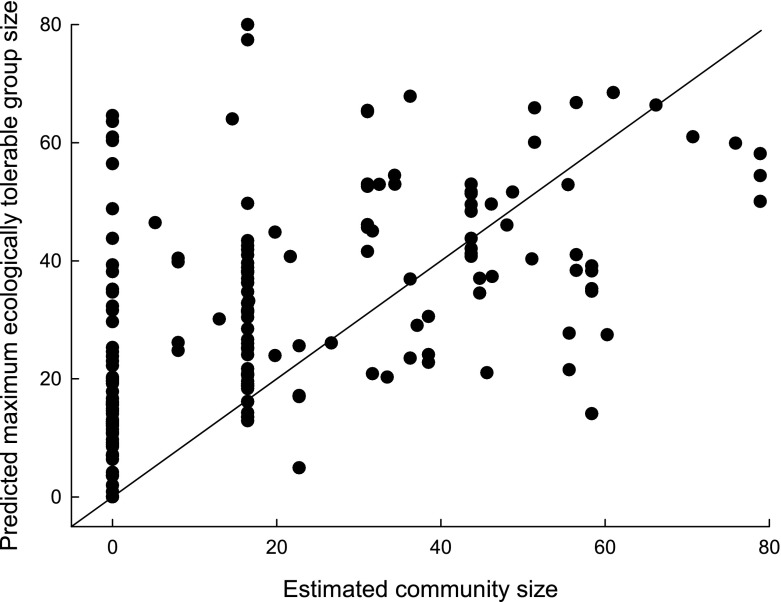
Modeled maximum ecologically tolerable group sizes (MEG) plotted against estimated community size for forest guenons across 202 sub-Saharan Africa sites; we estimated community size based on the sum of the mean group size of each species present at a site.

The time-budget model can be used to understand which time-budget components play the greatest role in shaping biogeography, group size, and community size. Predicted group sizes correlated positively with the observed species-richness and estimated community size at sites (excluding the nine sites for which the exact number of species present was not known: Spearman rank correlation *rs*_richness_= 0.62, *rs*_communitysize_= 0.62, *N* = 193, *P* < 0.001; using the subset of these sites where forest guenons are present: *rs* = 0.41, *rs* = 0.39, *N* = 119, *P* < 0.001; Fig. 4).

**Fig. 5:**
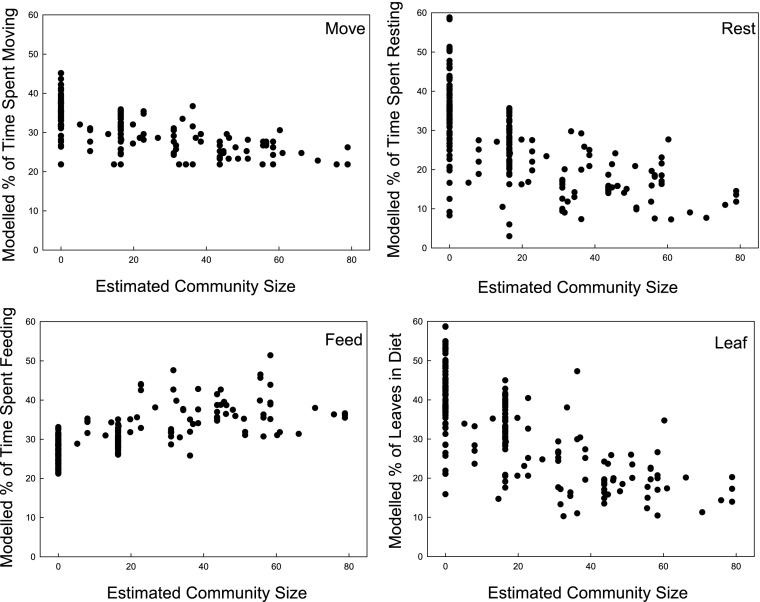
Percentage of daytime activity that forest guenon individuals are expected to spend on different activities according to a time-budget model plotted against estimated community size for 202 sub-Saharan African sites; group size is set to a fixed value of 20 and body mass to 4.4 kg.

Modeled moving and resting time and the percentage of leaf in the diet were significantly higher at the 74 sites where guenons are absent compared to the 128 presence sites (Mann–Whitney *U* test: *U*_rest_ = 1135.00, *U*_move_ = 1245.00, *U*_leaf_ = 1108.50, all *P* values < 0.001), indicating that high time requirements for these activities may prevent guenons from living at these sites. Modeled feeding-foraging time (calculated for a fixed group size of 20) was less at sites where guenons are absent (*U*_feed_ = 985.00, *P* < 0.001). Modeled feeding-foraging time correlated positively and modeled moving and resting times and percentage of leaves in the diet correlated negatively with estimated community size (Spearman rank correlation *rs*_feedforage_ = 0.762, *rs*_move_ = –0.709, *rs*_rest_ = –0.720, *rs*_leaf_ = –0.745, all *P* values < 0.001, at 193 sites for which number of species was known; *rs*_feedforage_ = 0.637, *rs*_move_ = –0.588, *rs*_rest_ = –0.573, *rs*_leaf_ = –0.648, *P* < 0.001, *N* = 119 excluding sites where guenons are absent; Fig. 5). This highlights that the limiting influence that feeding-foraging time had on group sizes was related not just to the competition between individuals but also to the conditions at the sites at which community sizes are larger. At those sites, individuals were predicted to spend more time feeding-foraging irrespective of group size itself (which we set to 20 in the equation in Fig. 5) as a result of the local climatic conditions.

## Discussion

Here we showed that time constraints were important determinants of the distribution and community size of forest guenons, but less important for limiting group size of species. We showed that presence/absence depended on enforced resting time, partly mediated by the percentage of leaf in the diet, and on moving time, partly mediated by body size. Resting and moving times at locations where forest guenons were absent were much higher than those where guenons were present. At sites where guenons were absent, there was not much time spared for feeding and socializing. Feeding-foraging time investment, in contrast, was lower at sites where forest guenons were absent compared to those where they were present. This shows that resting and moving, but not feeding-foraging time, constrains whether or not guenons can survive at a particular site. Feeding-foraging time, in contrast, was positively correlated with group size, suggesting that this sets the maximum achievable size for the guenon community at a site. The effect of group size on feeding foraging time, however, was not strong enough to explain why guenons live in groups as small as they typically do. According to the time constraints modeled here, guenon species should be able to live in larger groups.

### Environmental Determinants of Forest Guenon Distributions

Presence/absence of forest guenons was constrained mostly by mean annual temperature, temperature variation, and the relationship between rainfall and temperature (through moisture and growing season, P>2T) through their effects on resting and moving time, which is in line with the results of an ecological niche model for forest guenons (Korstjens 2018). Our analyses suggested that high levels of leaves in the diet (leading to high resting time values) and small body size (leading to high moving time values) can constrain guenon distribution patterns. In our pan-African analysis, we set body mass to a mean value of 4.4 kg to identify sites where a typical forest guenon could survive. The effects of body mass on moving time meant that larger species would achieve higher predicted group sizes than smaller species at the same site. Owing to the effect of the percentage of leaf in the diet on resting time, the model showed that species that consume less leaves could achieve larger group sizes than those consuming more leaves (all else being equal). Forest guenons do not have highly specialised adaptations for leaf digestion like colobines, but the overall pattern in primates is that larger species can depend more on leaves than smaller species because they have larger digestive systems (Fleagle 2013). Our analyses suggest that any species that is better at digesting leaves will require less resting time than a species that struggles with high percentages of leaf in the diet. This means that forest guenons with greater ability to survive on high levels of leaf in the diet can make use of a wider range of habitats than species that have less flexible digestive systems. In addition, moving time was negatively correlated to body size, providing larger species a greater flexibility in the types of environments they can occupy than smaller species. Indeed, most sites (*N* = 44 of 52) with just one guenon species contained *Cercopithecus mitis*, which is the largest species in the dataset and a species that has specialized adaptations to facilitate digestion of leaves (Bruorton *et al.* 1991). In addition, *C. mitis/albogularis* in South Africa shows significant negative correlations between “feeding time and temperature,” “leaf feeding and temperature,” and “leaf feeding and day length” (Coleman 2013; Ben Coleman *pers. comm*. 2017). This means that, in this population, *C. mitis* consumes more leaves in locations where, and time periods when, weather conditions are more challenging, i.e., during the wintertime or in colder areas of the home range. This suggests that their ability to cope with leaves and their large body size may allow *C. mitis* to occupy the most challenging environments for *Cercopithecus*.

The effect that leaf consumption has on time constraints is noteworthy because predicted climate change in several African areas will result in increased warming and reduced rainfall (Graham *et al.* 2016). Such changes will lead to lower quality of leaves as protein to fiber ratios will decrease (Korstjens and Dunbar 2007; Rothman *et al.* 2014) and concentrations of plant secondary compounds in leaves will increase (Dury *et al.* 1998; Rothman *et al.* 2014; Zvereva and Kozlov 2006). Reduced leaf quality will result in greater resting times and will likely make several locations that are currently suitable to forest guenons unsuitable under future conditions. We also showed that moving time is important in determining presence and absence of forest guenons. Both climate change and human forest modifications are likely to lead to increased travel times because they often result in forest fragmentation and forest degradation. As a result, animals will have to travel farther to find food sources that are more scattered and less abundant.

### Why Do Forest Guenons Live in Small Groups in Multispecies Communities?

Species-level group size was affected by intraspecific competition, evidenced by a positive group size effect on feeding-foraging time. However, the effect of group size on feeding-foraging time was weak: feeding-foraging time investment increased by only 0.39% with every individual added to the group. This suggests that the effect of increasing group size on feeding-foraging time was not enough to explain why forest guenons live in small groups within multispecies communities rather than larger single-species groups that outcompete other species. Moving time is also expected to increase with group size as a result of intraspecific competition as more individuals foraging together deplete food sources faster (Janson and van Schaik 1988). The guenon groups in our sample consumed both depletable food sources, fruits (53%) and flowers (9%), and nondepletable food sources, such as insects (17%) and leaves (19%). This suggests that there is a potential for food depletion leading to more travel but this effect was not detectable in the data sample. Our results are in line with those of other studies on competition in guenons: neither travel distance nor feeding time increases with group size in guenons when corrected for food availability (Cords 2012; Windfelder and Lwanga 2002).

Potential costs of large group sizes may be related to social strategies, as well as food competition (as modeled here). Previous work shows that primate species living in one-male–multifemale units do not necessarily show the expected increase in moving, feeding, and day-journey length with larger group sizes that multimale–multifemale units show (Snaith and Chapman 2005). It has been suggested that this is related to mating strategies, e.g., infanticide, being a social constraint on group size that acts by groups splitting up before the maximum ecologically tolerable group size has been reached (van Schaik 1996). For example, small group sizes in some colobine species and howlers have been linked to a strategy of limiting male coercion (in particular male infanticide and aggressive male group takeovers) and aggression, which can reduce female fitness (Dunbar and Dunbar 1988; Steenbeek *et al.* 2000; Treves and Chapman 1996). Infanticide occurs in guenons, often after a male takeover (Butynski 1990; Cords and Fuller 2010; Kane and Gnépa 2016; Struhsaker 1977). Larger groups may have a higher risk of male takeovers, but this requires further research in guenons.

## Conclusion

Our analyses showed that forest guenon biogeography is driven by constraints on minimum resting time and moving time. Resting and moving time in our sample increased with increasing body mass, higher proportion of leaves in the diet, higher temperatures, greater temperature variation, and shorter growing seasons. Climate change is likely to lead to greater increases in temperatures, temperature variation, and shorter growing seasons in many areas across Africa. This means that forest guenons will be negatively affected by predicted climate change. Feeding-foraging time increased with rainfall and group size, suggesting it sets the limit for community size. Because the effect of feeding-foraging on group size was small, feeding-foraging time constraints are not considered drivers of small group sizes in forest guenons.

## Electronic supplementary material

ESM 1 (DOCX 241 kb)
