# Supplementary material for: Time Constraints Do Not Limit Group Size in Arboreal Guenons but Do Explain Community Size and Distribution Patterns
Source: Int J Primatol. 2018 Jul 11;39(4):511–31. doi: 10.1007/s10764-018-0048-4 (PMC6182722; doi:10.1007/s10764-018-0048-4)
Supplement: Supplementary file 1 — (DOCX 241 kb) [file 10764_2018_48_MOESM1_ESM.docx]

**Electronic Supplementary Material to:**

**Time constraints do not limit group size in arboreal guenons but do explain community size and distribution patterns**

Amanda H. Korstjens**^*^**^a^, Julia Lehmann^b^, R.I.M. Dunbar^c^

^a^ Bournemouth University, Department of Life and Environmental Sciences, Poole, UK

^b^University of Roehampton, Department of Life Sciences, London, UK

^c^University of Oxford, Department of Experimental Psychology, Oxford, UK

**^*^ Corresponding author:** [akorstjens@bournemouth.ac.uk](mailto:akorstjens@bournemouth.ac.uk)

**Table SI Dietary information used to obtain the equation for percentage leaf in the diet**

Site codes are made up of 2-3 letters indicative of the country, and underscore, and an abbreviation for the site name and are consistent across data sets. Lat=Latitude; Lon=Longitude; percentage of feeding time spent feeding on: Leaf= vegetative matter, Fruit=fruit and seed, Flower=flower and flower buds, Fauna=invertebrates (mostly), Other includes lichen, fungi, moss; P_ann= average annual rainfall (mm); T_ann= average annual mean temperature (°C); T_mo_sd=standard deviation for monthly average temperature; Moi_mo_= average monthly moisture index; P>2T=productivity index.

| **Site** | **Species** | **Lat** | **Lon** | **Fruit** | **Leaf** | **Flower** | **Fauna** | **Other** | **P_ann** | **T_ann** | **T_mo_sd** | **Moi_mo_** | **P>2T** | **References** |
| --- | --- | --- | --- | --- | --- | --- | --- | --- | --- | --- | --- | --- | --- | --- |
| Ca_KL | *preussi* | 6.25 | 10.43 | 51.90 | 41.40 | 1.20 | 0.80 | 0.00 | 2910 | 18.70 | 1.06 | 0.200 | 9 | Beeson *et al.* 1996 |
| CI_Tai | *campbelli* | 6.17 | -3.67 | 46.30 | 8.40 | 1.00 | 33.10 | 10.90 | 1892 | 25.85 | 0.88 | -0.100 | 11 | Buzzard 2003 |
| CI_Tai | *diana* | 6.17 | -3.67 | 58.50 | 15.80 | 2.60 | 15.90 | 6.60 | 1892 | 25.85 | 0.88 | -0.100 | 11 | Buzzard 2003 |
| CI_Tai | *petaurista* | 6.17 | -3.67 | 33.60 | 39.70 | 6.20 | 12.30 | 7.50 | 1892 | 25.85 | 0.88 | -0.100 | 11 | Buzzard 2003 |
| DC_KB | *mitis* | -2.09 | 28.05 | 37.00 | 6.00 | 20.00 | 11.00 |  | 1800 | 19.70 | 0.24 | 0.315 | 11.5 | Schlichte 1978 |
| DC_Sal | *ascanius* | -1.25 | 22.37 | 67.70 | 16.50 | 15.70 |  |  | 1774 | 24.50 | 0.19 | 0.235 | 11 | Chapman *et al.* 2002 |
| Ga_Lop | *nictitans* | -0.53 | 11.54 | 70.40 | 16.00 | 9.90 | 3.10 | 0.00 | 1531 | 25.50 | 1.15 | 0.043 | 9 | Tutin *et al.* 1997 |
| Ga_Lop | *pogonias* | -0.53 | 11.54 | 77.90 | 6.50 | 9.10 | 6.50 | 0.00 | 1531 | 25.50 | 1.15 | 0.043 | 9 | Tutin *et al.* 1997 |
| Ga_Lop_Kla | *cephus* | -0.53 | 11.54 | 54.00 | 4.00 | 6.00 | 35.00 | 1.00 | 1531 | 25.50 | 1.15 | 0.043 | 9 | Tutin 1999 |
| Ga_Lop_Kla | *nictitans* | -0.53 | 11.54 | 48.00 | 17.00 | 9.00 | 24.00 | 2.00 | 1531 | 25.50 | 1.15 | 0.043 | 9 | Tutin 1999 |
| Ga_Mao | *cephus* | 0.57 | 12.87 | 78.10 | 6.00 | 0.50 | 12.60 |  | 1755 | 23.90 | 1.03 | 0.103 | 10 | Gautier-Hion 1980 |
| Ga_Mao | *nictitans* | 0.57 | 12.87 | 70.50 | 16.60 | 0.80 | 9.40 |  | 1755 | 23.90 | 1.03 | 0.103 | 10 | Gautier-Hion 1980 |
| Ga_Mao | *pogonias* | 0.57 | 12.87 | 82.50 | 1.20 | 0.10 | 16.10 |  | 1755 | 23.90 | 1.03 | 0.103 | 10 | Gautier-Hion 1980 |
| Ke_Kak | *ascanius* | 0.32 | 34.87 | 61.70 | 7.90 | 2.00 | 25.10 | 3.30 | 1859 | 22.00 | 0.67 | 0.320 | 11 | Cords 1986 |
| Ke_Kak | *mitis* | 0.32 | 34.87 | 57.10 | 20.00 | 3.70 | 16.80 | 2.40 | 1859 | 22.00 | 0.67 | 0.320 | 11 | Cords 1986 |
| Mw_Zom | *mitis* | -15.33 | 35.32 | 53.50 | 32.60 | 10.20 | 0.80 | 3.00 | 2014 | 23.20 | 2.41 | -0.143 | 7 | Beeson *et al.* 1996 |
| Rw_Nyu | *lhoesti* | -2.53 | 29.29 | 42.20 | 35.17 | 4.00 | 8.80 | 9.80 | 1744 | 15.25 | 0.54 | 0.110 | 11 | Kaplin & Moermond 2000 |
| Rw_Nyu | *mitis* | -2.53 | 29.29 | 56.70 | 6.20 | 6.40 | 24.90 | 6.20 | 1744 | 15.25 | 0.54 | 0.110 | 11 | Kaplin & Moermond 1998, 2000 |
| SA_CVi | *mitis* | -28.08 | 32.55 | 51.70 | 25.80 | 13.40 | 5.80 |  | 1155 | 21.50 | 2.55 | -0.120 | 10 | Lawes 1991 |
| SL_Tiw | *diana* | 7.31 | 11.20 | 42.75 | 12.55 | 16.10 | 27.65 |  | 2708 | 27.50 | 1.46 | 0.100 | 8 | Curtin 2002 |
| Ug_Bud | *mitis* | 1.73 | 31.55 | 50.80 | 29.35 | 10.00 | 9.65 |  | 1679 | 22.10 | 0.75 | -0.060 | 10.3 | Fairgrieve & Muhumuza 2003 |
| Ug_Kib_Dur | *ascanius* | 0.27 | 30.22 | 55.60 | 15.40 | 8.19 | 20.60 | 0.40 | 1703 | 20.18 | 0.90 | 0.080 | 11 | Chapman & Chapman 2000 |
| Ug_Kib_K30 | *mitis* | 0.57 | 30.35 | 22.05 | 31.35 | 6.38 | 39.42 | 0.80 | 1703 | 20.18 | 0.90 | 0.080 | 11 | Butynski 1990 |
| Ug_Kib_K30 | *ascanius* | 0.57 | 30.35 | 35.70 | 28.20 | 3.70 | 31.20 | 1.24 | 1703 | 20.18 | 0.90 | 0.080 | 11 | Chapman & Chapman 2000 |
| Ug_Kib_K30_70 | *mitis* | 0.57 | 30.35 | 45.13 | 20.90 | 11.75 | 19.83 | 2.00 | 1703 | 20.18 | 0.90 | 0.080 | 11 | Chapman & Chapman 2000 |
| Ug_Kib_Mai | *ascanius* | 0.27 | 30.25 | 59.70 | 12.90 | 11.60 | 14.50 | 1.36 | 1703 | 20.18 | 0.90 | 0.080 | 11 | Chapman & Chapman 2000 |
| Ug_Kib_Ngo | *mitis* | 0.50 | 30.42 | 30.10 | 22.80 | 9.80 | 35.90 | 1.30 | 1490 | 20.40 | 0.89 | 0.080 | 9.33 | Butynski 1990 |
| Ug_Kib_Seb | *ascanius* | 0.13 | 30.25 | 44.60 | 34.70 | 2.70 | 17.60 | 0.50 | 1703 | 20.18 | 0.90 | 0.080 | 11 | Chapman & Chapman 2000 |

**Table SII Group size data set used to compare observed and predicted group sizes and to estimate community size**

Climate data for these sites can be found in Table SIV following the site code. Site codes are made up of 2-3 letters indicative of the country, and underscore, and an abbreviation for the site name and are consistent across data sets. Lat=Latitude; Lon=Longitude; Indep gives indep when the site was chosen as one of our independent sites and appears in (Table SIV) but gives another site's name when that other site was chosen as the independent site because these sites were very close; climate information appears in Table SIIb for sites in Table SIIa that were considered not independent (indep) from another site; N_spp= number of forest guenon species at site; P_ann= average annual rainfall (mm); T_ann= average annual mean temperature (°C); T_mo_sd=standard deviation for monthly average temperature; Moimo= average monthly moisture index; P>2T=productivity index; N=average species-group size; MEG=maximum ecologically tolerable group size modeled for the site.

**Table SIIa. Group sizes (N) and predicted maximum ecologically tolerable group size (MEG)**

| **Site** | **Genus** | **Species** | **Indep** | **N_spp** | **N** | **MEG** | **References** |
| --- | --- | --- | --- | --- | --- | --- | --- |
| Ca_CM | *Cercopithecus* | *cephus* | indep | 4.00 | 12.00 | 46.07 | Mathews & Matthews 2002 |
| Ca_CM | *Cercopithecus* | *nictitans* | indep | 4.00 | 17.00 | 41.39 | Mathews & Matthews 2002 |
| Ca_CM | *Cercopithecus* | *pogonias* | indep | 4.00 | 16.00 | 46.55 | Mathews & Matthews 2002 |
| Ca_KL | *Allochrocebus* | *preussi* | Kimbi |  | 15.00 | 47.36 | Beeson *et al.* 1996 |
| Ca_MCa | *Allochrocebus* | *preussi* | Bimbia |  | 5.60 | 44.80 | Struhsaker 1969 |
| CAR_Ng | *Cercopithecus* | *cephus* | indep | 4.00 | 15.00 | 43.01 | Gautier-Hion *et al.* 1999 |
| CAR_Ng | *Cercopithecus* | *nictitans* | indep | 4.00 | 18.00 | 38.33 | Gautier-Hion *et al.* 1999 |
| CAR_Ng | *Cercopithecus* | *pogonias* | indep | 4.00 | 16.00 | 43.49 | Gautier-Hion *et al.* 1999 |
| CI_Tai | *Cercopithecus* | *campbelli* | indep | 4.00 | 9.00 | 44.51 | Buzzard 2004 |
| CI_Tai | *Cercopithecus* | *diana* | indep | 4.00 | 24.00 | 34.93 | Buzzard 2004 |
| CI_Tai | *Cercopithecus* | *petaurista* | indep | 4.00 | 11.00 | 37.08 | Buzzard 2004 |
| Co_Odz | *Cercopithecus* | *cephus* | indep | 4.00 | 9.30 | 53.65 | Gautier-Hion *et al.* 1999 |
| Co_Odz | *Cercopithecus* | *neglectus* | indep | 4.00 | 8.00 | 49.94 | Gautier-Hion *et al.* 1999 |
| Co_Odz | *Cercopithecus* | *nictitans* | indep | 4.00 | 14.60 | 48.98 | Gautier-Hion *et al.* 1999 |
| Co_Odz | *Cercopithecus* | *pogonias* | indep | 4.00 | 16.00 | 54.13 | Gautier-Hion *et al.* 1999 |
| DC_Ike | *Cercopithecus* | *ascanius* | indep |  | 10.00 | 55.63 | Mate, *et al.* 1995 |
| DC_Ike | *Cercopithecus* | *neglectus* | indep |  | 3.00 | 52.15 | Mate *et al.* 1995 |
| DC_Ike | *Cercopithecus* | *wolfi* | indep |  | 12.00 | 55.99 | Mate *et al.* 1995 |
| DC_Itu | *Cercopithecus* | *ascanius* | indep | 6.00 | 4.00 | 61.94 | Thomas 1991 |
| DC_Itu | *Cercopithecus* | *denti* | indep | 6.00 | 9.60 | 62.06 | Thomas 1991 |
| DC_Itu | *Cercopithecus* | *hamlyni* | indep | 6.00 | 2.00 | 59.55 | Thomas 1991 |
| DC_Itu | *Allochrocebus* | *lhoesti* | indep | 6.00 | 2.00 | 59.16 | Thomas 1991 |
| DC_Itu | *Cercopithecus* | *mitis* | indep | 6.00 | 4.00 | 57.15 | Thomas 1991 |
| DC_Itu | *Cercopithecus* | *neglectus* | indep | 6.00 | 3.00 | 58.47 | Thomas 1991 |
| DC_KB | *Cercopithecus* | *ascanius* | indep | 5.00 | 10.10 | 63.02 | Hall *et al.* 2003 |
| DC_KB | *Cercopithecus* | *denti* | indep | 5.00 | 9.80 | 63.14 | Hall *et al.* 2003 |
| DC_KB | *Cercopithecus* | *hamlyni* | indep | 5.00 | 2.70 | 60.62 | Hall *et al.* 2003 |
| DC_KB | *Cercopithecus* | *mitis* | indep | 5.00 | 12.50 | 58.23 | Schlichte 1978; Lawes, *et al.* 1990 |
| DC_Lom | *Cercopithecus* | *ascanius* | indep | 3.00 | 23.00 | 54.96 | McGraw 1994 |
| DC_Lom | *Cercopithecus* | *neglectus* | indep | 3.00 | 5.00 | 51.49 | McGraw 1994 |
| DC_Lom | *Cercopithecus* | *wolfi* | indep | 3.00 | 10.10 | 55.32 | McGraw 1994 |
| DC_Sal | *Cercopithecus* | *wolfi* | indep | 3.00 | 16.00 | 56.85 | Gautier-Hion 2013 |
| DC_Wam | *Cercopithecus* | *dryas* | indep | 4.00 | 23.00 | 57.18 | Butynski 2013a |
| EG_Bio | *Cercopithecus* | *erythrotis* | Bimbia | 4.00 | 10.00 | 39.64 | Butynski & Kingdon 2013 |
| EG_Bio | *Allochrocebus* | *preussi* | Bimbia | 4.00 | 7.50 | 37.00 | Butynski 2013b |
| Et_Sha | *Cercopithecus* | *cephus* | indep | 4.00 | 10.00 | 69.05 | White 1994 |
| Et_Sha | *Cercopithecus* | *nictitans* | indep | 4.00 | 14.00 | 64.38 | White 1994 |
| Et_Sha | *Cercopithecus* | *pogonias* | indep | 4.00 | 13.00 | 69.53 | White 1994 |
| Ga_Fab | *Cercopithecus* | *nictitans* | indep | 4.00 | 11.00 | 35.97 | Brugiere *et al.* 2002 |
| Ga_Lop | *Cercopithecus* | *cephus* | ForetAbeil | 4.00 | 13.00 | 39.17 | Tutin 1999 |
| Ga_Lop | *Cercopithecus* | *nictitans* | ForetAbeil | 4.00 | 10.00 | 34.49 | Tutin 1999 |
| Ga_Lop | *Cercopithecus* | *pogonias* | ForetAbeil | 4.00 | 12.60 | 39.64 | Tutin 1999 |
| Ga_Maa | *Cercopithecus* | *cephus* | indep | 4.00 | 10.00 | 43.31 | Brugiere *et al.* 2002; Chapman *et al.* 2002 |
| Ga_Maa | *Cercopithecus* | *nictitans* | indep | 4.00 | 11.00 | 38.64 | Brugiere *et al.* 2002; Chapman *et al.* 2002 |
| Ga_Maa | *Cercopithecus* | *pogonias* | indep | 4.00 | 14.00 | 43.79 | Brugiere *et al.* 2002; Chapman *et al.* 2002 |
| Ga_Maa | *Allochrocebus* | *solatus* | indep | 4.00 | 18.00 | 38.52 | Brugiere *et al.* 2002; Chapman *et al.* 2002 |
| Ga_Mao | *Cercopithecus* | *cephus* | Makande | 4.00 | 2.35 | 48.02 | Chapman *et al.* 2002 |
| Gh_BF | *Cercopithecus* | *lowei* | indep | 1.00 | 16.60 | 35.95 | Galat-Luong *et al.* 2013 |
| Gh_Bia | *Cercopithecus* | *roloway* | Tai | 3.00 | 17.50 | 34.92 | Curtin 2002 |
| Ke_Elg | *Cercopithecus* | *neglectus* | Kakamega | 2.00 | 7.00 | 62.23 | Mugambi *et al.* 1997 |
| Ke_Kak | *Cercopithecus* | *ascanius* | indep | 3.00 | 21.00 | 69.90 | Fashing & Cords 2000; Chapman *et al.* 2002 |
| Ke_Kak | *Cercopithecus* | *mitis* | indep | 3.00 | 33.00 | 65.10 | Fashing & Cords 2000 |
| Ke_Kis | *Cercopithecus* | *mitis* | indep | 1.00 | 6.00 | 35.50 | Moreno-Black & Maples 1977; Lawes *et al.* 1990 |
| Ke_Nya | *Cercopithecus* | *mitis* | Aberdare |  | 16.00 | 61.44 | de Vos & Omar 1971; Lawes *et al.* 1990 |
| Ke_TR | *Cercopithecus* | *mitis* | indep | 1.00 | 17.00 | 27.65 | Karere *et al.* 2004 |
| Rw_Nyu | *Cercopithecus* | *hamlyni* | Kibira | 5.00 | 24.00 | 65.49 | Hart *et al.* 2013 |
| Rw_Nyu | *Allochrocebus* | *lhoesti* | Kibira | 5.00 | 28.00 | 65.10 | Kaplin 2001 |
| Rw_Nyu | *Cercopithecus* | *mitis* | Kibira | 5.00 | 29.00 | 63.09 | Kaplin 2001 |
| SA_CVi | *Cercopithecus* | *mitis* | indep | 1.00 | 33.00 | 34.21 | Henzi & Lawes 1987; Butynski 1990; Payne, *et al.* 2003 |
| SA_Doo | *Cercopithecus* | *mitis* | indep | 1.00 | 10.00 | 17.83 | Lawes *et al.* 1990 |
| SA_Hlu | *Cercopithecus* | *mitis* | Itala | 1.00 | 23.00 | 32.05 | Wolfheim 1983 |
| SA_Ngo | *Cercopithecus* | *mitis* | Cape Vidal | 1.00 | 17.00 | 35.26 | Lawes *et al.* 1990 |
| SL_OK | *Cercopithecus* | *campbelli* | Loma-Mts | 2.00 | 7.70 | 21.21 | Galat *et al.* 2013 |
| SL_Tiw | *Cercopithecus* | *campbelli* | CapMount | 3.00 | 14.00 | 20.36 | Oates *et al.* 1990 |
| SL_Tiw | *Cercopithecus* | *diana* | CapMount | 3.00 | 20.00 | 18.08 | Oates *et al.* 1990 |
| SL_Tiw | *Cercopithecus* | *petaurista* | CapMount | 3.00 | 14.00 | 20.24 | Oates *et al.* 1990 |
| SP_STo | *Cercopithecus* | *mona* | indep | 1.00 | 8.00 | 42.04 | Glenn, *et al.* 2002 |
| Ta_Mah | *Cercopithecus* | *ascanius* | indep | 2.00 | 15.00 | 47.67 | Uehara 2003 |
| Ta_Mah | *Cercopithecus* | *mitis* | indep | 2.00 | 10.00 | 42.87 | Uehara 2003 |
| Ta_Udz | *Cercopithecus* | *mitis* | Mikumi |  | 8.00 | 30.79 | Dinesen *et al.* 2001 |
| Ug_Bud | *Cercopithecus* | *mitis* | indep | 2.00 | 14.00 | 50.21 | Aldrich-Blake 1970 |
| Ug_Kal | *Allochrocebus* | *lhoesti* | Virunga | 3.00 | 34.00 | 62.78 | Tashiro 2006 |
| Ug_Kal | *Cercopithecus* | *mitis* | Virunga | 3.00 | 20.00 | 60.77 | Tashiro 2006 |
| Ug_Kib | *Cercopithecus* | *ascanius* | indep | 3.00 | 19.00 | 67.92 | Butynski 1990 |
| Ug_Kib | *Allochrocebus* | *lhoesti* | indep | 3.00 | 17.50 | 65.14 | Sarmiento 2013 |
| Ug_Kib | *Cercopithecus* | *mitis* | indep | 3.00 | 16.49 | 63.13 | Butynski 1990 |

**Table SIIb: Climate information for those sites in Table SIIa that are not included in Table SIV**

| **Site** | **Site name** | **Country** | **Lat** | **Lon** | **P_ann** | **T_ann** | **T_mo_sd** | **Moi_mo_** | **P>2T** |
| --- | --- | --- | --- | --- | --- | --- | --- | --- | --- |
| Ke_Nya | NyamweruKikuyu | Kenya | -0.88 | 36.71 | 1047 | 17.50 | 1.10 | 0.02 | 10.25 |
| Ca_MCa | MntCameroon | Cameroon | 4.12 | 9.17 | 2366 | 19.20 | 0.86 | 0.26 | 9.50 |
| EG_Bio | Bioko | Equatorial Guinea | 3.33 | 8.55 | 1900 | 24.60 | 0.86 | 0.09 | 9.00 |
| SA_Ngo | Ngoye | South Africa | -28.85 | 31.63 | 1034 | 19.87 | 2.72 | -0.01 | 9.00 |
| SL_Tiw | Tiwai | Sierra Leone | 7.52 | -11.33 | 2839 | 26.05 | 0.90 | 0.10 | 8.50 |
| Ga_Lop | Lope | Gabon | -0.57 | 11.56 | 1761 | 23.88 | 1.15 | 0.04 | 9.00 |
| SA_Hlu | Hluhluwe-Umfolozi | South Africa | -28.22 | 31.93 | 950 | 21.48 | 2.63 | -0.16 | 9.00 |
| Ke_Elg | Mt-Elgon | Kenya | 1.11 | 34.65 | 1321 | 20.25 | 0.78 | 0.18 | 11.25 |
| Rw_Nyu | Nyungwe | Rwanda | -2.53 | 29.29 | 1166 | 18.80 | 0.38 | 0.11 | 9.25 |
| Ca_KL | Kilum-Ljim | Cameroon | 6.25 | 10.43 | 1950 | 18.70 | 1.06 | 0.20 | 9.00 |
| SL_OK | Outamba-Kilimi | Sierra Leone | 9.75 | -12.04 | 2068 | 26.75 | 1.32 | -0.12 | 8.00 |
| Ga_Mao | Makokou | Gabon | 0.57 | 12.87 | 1645 | 23.35 | 0.96 | 0.10 | 10.00 |
| Ta_Udz | Udzungwas | Tanzania | -7.80 | 36.66 | 1374 | 21.15 | 1.76 | -0.12 | 7.25 |
| Gh_Bia | Bia | Ghana | 6.57 | -3.07 | 1440 | 25.75 | 1.19 | -0.11 | 9.00 |
| Ug_Kal | Kalinzu | Uganda | -0.38 | 30.10 | 1039 | 20.98 | 0.33 | -0.02 | 9.75 |

**Table SIII** **Details for the studies with time budget data; estimated community size (N_comm_); species-group size (N), percentage of time spent feeding-foraging (Feed+Forg), moving (Move), resting (Rest), and social (Social), percentage of feeding time spent feeding on leaves (Leaf), average annual rainfall (P_ann in mm) and temperature (T_ann, in °C), standard deviation for monthly average temperature (T_mo_sd), and average monthly (Moi_mo_) moisture index, productivity (P>2T), number of forest guenon species at site (N_spp), average body weight of adult female/ male (BM_afam)**

| **Country** | **Site** | **Species** | **N_comm_** | **N** | **Feed+ Forg** | **Rest** | **Move** | **Social** | **Leaf** | **P_ann** | **T_ann** | **T_mo_sd** | **Moi_mo_** | **P>2T** | **N_spp** | **BM_afam** |
| --- | --- | --- | --- | --- | --- | --- | --- | --- | --- | --- | --- | --- | --- | --- | --- | --- |
| Rwanda^1^ | Nyungwe | *A. lhoesti* | 70.71 | 27.50 | 47.60 | 19.40 | 22.70 | 10.4 | 35.17 | 1744.0 | 15.25 | 0.54 | 0.11 | 11.00 | 5 | 4.71 |
| Rwanda^1^ | Nyungwe | *C. mitis* | 70.71 | 29.00 | 49.20 | 20.40 | 20.40 | 11.4 | 6.20 | 1744.0 | 15.25 | 0.54 | 0.11 | 11.00 | 5 | 5.55 |
| Ivory Coast^2^ | Taï NP | *C. diana* | 58.39 | 23.50 | 40.60 | 24.40 | 27.70 | 7.3 | 15.80 | 1891.8 | 25.85 | 0.88 | -0.10 | 11.00 | 4 | 4.55 |
| Ivory Coast^2^ | Taï NP | *C. campbelli* | 58.39 | 9.00 | 48.50 | 18.00 | 28.00 | 5.2 | 8.40 | 1891.8 | 25.85 | 0.88 | -0.10 | 11.00 | 4 | 3.60 |
| Ivory Coast^2^ | Taï NP | *C. petaurista* | 58.39 | 13.00 | 45.20 | 23.80 | 25.60 | 5.4 | 39.70 | 1891.8 | 25.85 | 0.88 | -0.10 | 11.00 | 4 | 3.65 |
| Kenya^3^ | Kakamega | *C. mitis* | 36.25 | 43.00 | 54.00 | 36.10 | 9.00 | 1.4 | 20.00 | 1859.0 | 22.00 | 0.67 | 0.32 | 11.00 | 3 | 5.55 |
| Kenya^3^ | Kakamega | *C. ascanius* | 36.25 | 22 | 38.50 | 21.00 | 38.00 | 1.25 | 7.9 | 1859.0 | 22.00 | 0.67 | 0.32 | 11.00 | 3 | 3.55 |
| Sierra Leone^4^ | Tiwai Island | *C. diana* | 44.73 | 30.50 | 35.00 | 29.00 | 25.00 | 6.0 | 12.55 | 2708.0 | 27.50 | 1.46 | 0.10 | 8.00 | 3 | 4.55 |
| Uganda^5^ | Budongo | *C. mitis* | 31.05 | 14.00 | 41.70 | 39.40 | 16.50 | 2.4 | 29.35 | 1678.5 | 22.10 | 0.75 | -0.06 | 10.30 | 2 | 5.55 |
| Uganda^6^ | Kanyawara^10^ | *C. ascanius* | 51.43 | 32.50 | 34.10 | 30.60 | 17.40 | 7.4 | 16.10 | 1702.9 | 20.18 | 0.90 | 0.08 | 11.00 | 3 | 3.55 |
| Uganda^6^ | Kanyawara^10^ | *C. mitis* | 51.43 | 24.00 | 38.10 | 33.10 | 16.50 | 6.1 | 20.90 | 1702.9 | 20.18 | 0.90 | 0.08 | 11.00 | 3 | 5.55 |
| Uganda^7^ | Kanyawara^10^ | *C. mitis* | 51.43 | 18.00 | 36.23 | 35.68 | 19.73 | 8.33 | 20.90 | 1702.9 | 20.18 | 0.90 | 0.08 | 11.00 | 3 | 5.55 |
| Uganda^6^ | Ngogo^10^ | *C. mitis* | 51.43 | 23.00 | 31.70 | 36.2 | 24.7 | 7.0 | 22.80 | 1490.0 | 20.40 | 0.89 | 0.08 | 9.33 | 3 | 5.55 |
| Uganda^8^ | Kalinzu | *A. lhoesti* | 51.43 | 30.50 | 32.80 | 31.07 | 28.58 | 7.91 | 27.24 | 1116.2 | 20.98 | 0.33 | 0.01 | 9.75 | 3 | 4.71 |
| South Africa^9^ | Cape Vidal | *C. mitis* | 16.46* | 32.50 | 35.80 | 22.60 | 29.40 | 8.0 | 25.80 | 1155.0 | 21.50 | 2.55 | -0.12 | 10.00 | 1 | 5.55 |

Notes: *Average group size of *C. mitis* (i.e. Ncomm in this location) is 16.46 but at Cape Vidal group sizes are much larger. 1. Kaplin & Moermond 1998, 2000, 2. Buzzard 2004 3.Cords 1986, 1989; Chapman *et al.* 2002, 4. Whitesides 1989; Hill & Oates 2013, 5. Lawes, *et al.* 1990; Fairgrieve & Muhumuza 2003; time budgets from Aldrich-Blake 1970 PhD thesis cited in Lawes *et al.* 1990, 6. Butynski 1990, 7. Struhsaker & Leland 1979: citing Rudran Pers. Comm., 8. Tolo, *et al.* 2008, 9. Lawes, *et al.* 1990; Lawes 1991. ^10^ Kanyawara and Ngogo are both in Kibale National Park, Uganda, but separated. The two values for Kibale Kanyawara, *C. mitis*, stem from two separate studies, one done in the 70s and one recent study, which is why these are entered separately.

**Table SIV Climate and guenon community data for each of the 202 independent African sites used.**

Site codes are made up of 2-3 letters indicative of the country, and underscore, and an abbreviation for the site name and are consistent across data sets. Lat'=Latitude; Lon=Longitude; Guens= presence of forest guenons, 1=present, 0=absent; N_spp= number of forest guenon species at site; P_ann= average annual rainfall (mm); T_ann= average annual mean temperature (°C); T_mo_sd=standard deviation for monthly average temperature; Moimo= average monthly moisture index; P>2T=productivity index; MEG= modeled maximum ecologically tolerable group size.

| **Site** | **Site name** | **Country** | **Lat** | **Lon** | **Guens** | **N_spp** | **P_ann** | **T_ann** | **T_mo_sd** | **Moi_mo_** | **P>2T** | **MEG** | **References** |
| --- | --- | --- | --- | --- | --- | --- | --- | --- | --- | --- | --- | --- | --- |
| An_Bik | Bikuar | Angola | -15.29 | 14.81 | 0 | 0 | 726 | 21.83 | 2.80 | -0.468 | 6.25 | 22.22 | WCMC & http://www.wildlifesafari.info |
| An_Buf | Bufalo | Angola | -12.78 | 13.81 | 0 | 0 | 864 | 22.95 | 1.77 | -0.400 | 6.75 | 31.61 | APAAT & Ministério do urbanismo e ambiente 2006 |
| An_Ion | Iona | Angola | -16.64 | 12.33 | 0 | 0 | 209 | 21.40 | 1.89 | -0.820 | 1.50 | 20.26 | APAAT & WCMC |
| An_Kam | Kameia | Angola | -11.91 | 21.64 | 0 | 0 | 1272 | 21.43 | 1.62 | -0.153 | 7.00 | 34.70 | WCMC & http://www.wildlifesafari.info |
| An_Kan | Kangandala | Angola | -9.87 | 16.73 | 1 | 2 | 1188 | 21.50 | 0.88 | -0.100 | 7.50 | 46.12 | WCMC & http://www.wildlifesafari.info |
| An_Kis | Kisama | Angola | -9.75 | 13.62 | 1 | 1 | 771 | 25.10 | 2.18 | -0.540 | 5.50 | 18.32 | WCMC & http://www.wildlifesafari.info |
| An_Lua | Luando | Angola | -11.11 | 17.62 | 1 | 2 | 1277 | 21.43 | 1.20 | -0.095 | 7.50 | 41.58 | APAAT |
| An_Lui | Luiana | Angola | -17.35 | 22.69 | 0 | 0 | 625 | 22.20 | 3.10 | -0.555 | 5.00 | 14.53 | APAAT |
| An_Mav | Mavinga | Angola | -15.50 | 20.93 | 0 | 0 | 867 | 21.13 | 2.98 | -0.403 | 5.00 | 16.65 | APAAT |
| Be_Ala | Alafiarou | Benin | 9.02 | 2.40 | 1 | 1 | 1169 | 26.25 | 1.57 | -0.280 | 7.25 | 26.14 | Campbell *et al.* 2008 |
| Be_Lam | Lama | Benin | 6.96 | 2.15 | 1 | 2 | 1102 | 27.05 | 1.29 | -0.333 | 8.00 | 30.11 | Campbell *et al.* 2008 |
| Be_PNW | PN de West | Benin | 11.89 | 2.71 | 0 | 0 | 835 | 27.30 | 2.32 | -0.560 | 5.00 | 9.68 | Centre d’echange d’information du Benin *2004* |
| BF_DBa | Deux Bales | Burkina-Faso | 11.60 | -2.94 | 0 | 0 | 931 | 27.63 | 2.12 | -0.498 | 5.00 | 10.71 | WCMC |
| BF_KTa | Kabore-Tambi | Burkina-Faso | 11.48 | -1.26 | 0 | 0 | 849 | 26.75 | 1.90 | -0.528 | 5.00 | 15.56 | WCMC |
| Bo_Cho | Chobe | Botswana | -18.54 | 24.51 | 0 | 0 | 717 | 21.05 | 3.41 | -0.493 | 5.00 | 13.05 | WCMC & http://www.wildlifesafari.info |
| Bo_Mak | Makgadikgadi | Botswana | -20.61 | 24.78 | 0 | 0 | 390 | 21.58 | 4.53 | -0.738 | 4.25 | .00 | WCMC & http://www.wildlifesafari.info |
| Bo_Mor | Moremi | Botswana | -19.28 | 23.21 | 0 | 0 | 493 | 22.00 | 3.88 | -0.658 | 5.00 | 6.96 | WCMC & http://www.wildlifesafari.info |
| Bu_Bur | Bururi | Burundi | -3.94 | 29.60 | 1 | 2 | 1218 | 21.00 | 0.63 | -0.040 | 8.25 | 52.60 | WCMC & APAAT |
| Bu_Kib | Kibira | Burundi | -2.92 | 29.43 | 1 | 3 | 1115 | 20.18 | 0.41 | -0.015 | 8.75 | 60.06 | Nzigidahera 2000 |
| Ca_Ben | Benoue | Cameroon | 8.35 | 13.83 | 0 | 0 | 1177 | 27.60 | 1.93 | -0.405 | 6.75 | 16.03 | WCMC |
| Ca_Bim | Bimbia | Cameroon | 4.02 | 9.24 | 1 | 3 | 2366 | 19.20 | 0.86 | 0.260 | 9.50 | 45.04 | Glenn *et al.* 2002 |
| Ca_CM | CampMa'an | Cameroon | 2.51 | 10.32 | 1 | 4 | 2237 | 24.53 | 0.85 | 0.218 | 12.00 | 43.79 | WCMC & APAAT Colyn & Deleporte 2002; Mathews & Matthews 2002; Matthews 2004 |
| Ca_Dja | Dja | Cameroon | 3.03 | 13.00 | 1 | 4 | 1607 | 23.35 | 0.65 | 0.123 | 9.50 | 48.37 | Colyn & Deleporte 2002; Gautier-Hion & Brugière 2005 & WCMC |
| Ca_Ebo | Ebo | Cameroon | 4.31 | 10.34 | 1 | 5 | 2688 | 25.68 | 1.10 | 0.140 | 9.00 | 21.53 | *http://www.berggorilla.de/english/gjournal/texte/28ebo.html & Morgan, et al. 2003* |
| Ca_Kal | Kalamaloue | Cameroon | 12.13 | 14.89 | 0 | 0 | 559 | 27.90 | 3.07 | -0.703 | 3.50 | .00 | APAAT |
| Ca_Kim | Kimbi | Cameroon | 6.59 | 10.38 | 1 | 4 | 1938 | 22.40 | 1.04 | 0.080 | 8.50 | 37.33 | APAAT |
| Ca_Kor | Korup | Cameroon | 5.22 | 8.96 | 1 | 5 | 2780 | 24.25 | 0.91 | 0.215 | 9.50 | 27.71 | Colyn & Deleporte 2002; Waltert *et al.* 2002 & Waltert pers. comm. |
| Ca_Lob | Lobeke | Cameroon | 2.30 | 15.89 | 1 | 4 | 1584 | 24.65 | 0.62 | 0.088 | 11.25 | 51.43 | APAAT & Cameroon List pers. comm. |
| Ca_MD | MbamDjerem | Cameroon | 5.85 | 12.78 | 1 | 2 | 1603 | 23.43 | 0.88 | -0.005 | 8.50 | 40.72 | WCMC & APAAT |
| Ca_MG | Mozogo-Gokoro | Cameroon | 10.96 | 13.93 | 0 | 0 | 823 | 26.25 | 2.31 | -0.535 | 5.00 | 12.41 | WCMC & APAAT |
| Ca_Bou | Boumba | Cameroon | 2.51 | 14.72 | 1 | 4 | 1585 | 24.00 | 0.63 | 0.113 | 11.25 | 52.97 | WCMC & APAAT |
| CAR_MG | Manova-Gounda | Central African | 8.91 | 21.39 | 1 | 3 | 1053 | 26.78 | 1.63 | -0.410 | 6.00 | 20.28 | Colyn & Deleporte 2002; WCMC |
| CAR_Ng | Ngotto | Central African | 3.96 | 17.01 | 1 | 4 | 1510 | 25.03 | 0.82 | -0.035 | 9.00 | 40.73 | Brugière, *et al.* 1999a, 199b; Gautier-Hion & Brugière 2005 |
| CAR_VB | Vassako-Bolo | Central African | 8.15 | 19.77 | 1 | 2 | 1194 | 26.93 | 1.39 | -0.338 | 6.75 | 23.91 | WCMC & APAAT |
| CI_Bol | Bolo | Côte d'Ivoire | 5.27 | -5.92 | 1 | 3 | 1561 | 25.60 | 0.98 | -0.083 | 10.25 | 40.30 | Sery *et al.* 2006; Gonedelé Bi *et al.* 2012 |
| CI_Cav | Cavally | Côte d'Ivoire | 6.17 | -7.79 | 1 | 4 | 1958 | 26.13 | 1.22 | 0.080 | 10.75 | 34.83 | Alonso, *et al.* 2005 |
| CI_Com | Comoe | Côte d'Ivoire | 9.12 | -3.73 | 1 | 4 | 1113 | 26.53 | 1.49 | -0.320 | 7.50 | 27.45 | Fischer *et al.* 1999; WCMC |
| CI_Ehi | Ehi | Côte d'Ivoire | 5.15 | -2.92 | 1 | 3 | 1948 | 26.05 | 1.06 | -0.020 | 10.50 | 34.53 | McGraw 1998, 2005 |
| CI_Hdo | HauteDodo | Côte d'Ivoire | 4.90 | -7.32 | 1 | 4 | 2499 | 25.25 | 0.96 | 0.338 | 12.00 | 39.14 | Alonso *et al.* 2005 |
| CI_Mar | Marahoue | Côte d'Ivoire | 7.11 | -6.02 | 1 |  | 1265 | 25.60 | 1.02 | -0.190 | 9.50 | 40.31 | WCMC |
| CI_Nim | Nimba | Côte d'Ivoire | 7.57 | -8.43 | 1 | 4 | 2007 | 24.93 | 0.73 | 0.060 | 9.75 | 38.27 | WCMC & APAAT |
| CI_Tai | Tai | Côte d'Ivoire | 6.17 | -3.67 | 1 | 4 | 1554 | 26.13 | 1.19 | -0.100 | 9.75 | 35.28 | McGraw, *et al.* 2007 |
| CO_Lef | Lefini | Congo Republic | -2.96 | 15.42 | 1 | 4 | 1809 | 23.23 | 0.75 | 0.170 | 9.75 | 46.04 | Mathot, *et al.* 2006; King 2008 |
| CO_Lik | Likouala | Congo Republic | 1.41 | 17.80 | 1 | 4 | 1751 | 24.95 | 0.63 | 0.158 | 12.00 | 51.66 | Blake 1993 |
| Co_Odz | Odzala | Congo Republic | 0.77 | 14.83 | 1 | 4 | 1595 | 24.38 | 0.67 | 0.095 | 11.25 | 51.37 | Bermejo 1999; Gautier-Hion & Brugière 2005 |
| DC_Bon | Bondo | DRCongo | 3.82 | 23.67 | 1 | 4 | 1696 | 24.85 | 0.61 | 0.095 | 11.25 | 49.58 | Gevaerts 1992 |
| DC_Gar | Garamba | DRCongo | 4.16 | 29.50 | 1 | 2 | 1549 | 23.80 | 0.82 | 0.063 | 9.25 | 44.84 | WCMC & APAAT; Rowcliffe, de Merode & Cowlishaw 2004 |
| DC_Ike | Ikela | DRCongo | -1.12 | 23.52 | 1 |  | 1916 | 25.38 | 0.21 | 0.205 | 12.00 | 53.59 | Mate *et al.* 1995 |
| DC_Itu | Ituri | DRCongo | 1.71 | 28.55 | 1 | 6 | 1683 | 23.33 | 0.36 | 0.233 | 12.00 | 59.91 | Thomas 1991 |
| DC_KB | Kahuzi-Biega | DRCongo | -2.10 | 28.16 | 1 | 5 | 1861 | 21.93 | 0.24 | 0.315 | 11.50 | 60.98 | Convention sur la Diversite Biologique |
| DC_Kin | Kindu | DRCongo | -2.92 | 25.92 | 1 | 6 | 1594 | 23.98 | 0.40 | 0.073 | 9.75 | 50.04 | Gevaerts 1992 |
| DC_Kis | Kisangani | DRCongo | 0.52 | 25.52 | 1 | 6 | 1669 | 24.68 | 0.53 | 0.153 | 12.00 | 54.42 | Gevaerts 1992 |
| DC_Kun | Kundelungu | DRCongo | -10.08 | 27.41 | 1 | 1 | 1083 | 21.55 | 1.60 | -0.230 | 7.00 | 36.31 | Convention sur la Diversite Biologique |
| DC_Lok | Lokutu | DR Congo | 1.15 | 23.61 | 1 | 3 | 1799 | 24.85 | 0.49 | 0.178 | 12.00 | 52.95 | Butynski & McCullough 2007 |
| DC_Lom | Lomako | DRCongo | 0.08 | 21.08 | 1 | 3 | 2059 | 24.40 | 0.45 | 0.343 | 12.00 | 52.92 | McGraw 1994 |
| DC_Luk | Lukuru | DRCongo | -3.75 | 21.35 | 1 |  | 1578 | 24.20 | 0.47 | 0.080 | 10.00 | 49.99 | Thompson 1997 |
| DC_Mai | Maiko | DRCongo | -0.44 | 26.67 | 1 | 6 | 1714 | 23.53 | 0.46 | 0.243 | 12.00 | 58.13 | Convention sur la Diversite Biologique |
| DC_Sal | Salonga | DRCongo | -2.36 | 20.97 | 1 | 3 | 1831 | 24.48 | 0.41 | 0.203 | 12.00 | 54.45 | Chapman *et al.* 1999; Amy Cobden pers. comm. |
| DC_Upe | Upemba | DRCongo | -9.06 | 26.64 | 1 | 3 | 1207 | 23.35 | 1.10 | -0.190 | 7.00 | 36.91 | Convention sur la Diversite Biologique |
| 5 | Virunga | DRCongo | -0.34 | 29.41 | 1 | 3 | 1147 | 20.55 | 0.35 | 0.075 | 10.50 | 66.36 | Owiunji *et al.* 2005; APAAT |
| DC_Wam | Wamba | DRCongo | 0.18 | 22.47 | 1 | 4 | 2065 | 25.10 | 0.24 | 0.288 | 12.00 | 52.87 | Kano 1996 |
| EG_Mat | Matama | Equatorial Guin | 1.47 | 10.50 | 1 | 4 | 2239 | 23.98 | 0.87 | 0.255 | 11.00 | 42.07 | Gautier-Hion *et al.* 1999 |
| Et_Awa | Awash | Ethiopia | 8.91 | 40.03 | 0 | 0 | 885 | 21.53 | 1.68 | -0.300 | 7.25 | 38.15 | http://www.selamta.net/awash_national_park.htm; & WCMC |
| Et_Bal | Bale | Ethiopia | 6.97 | 39.69 | 1 | 1 | 886 | 11.40 | 0.71 | 0.103 | 10.50 | 79.99 | http://www.selamta.net/bale_mountains_national_park.htm & WCMC |
| Et_Bol | Bole | Ethiopia | 9.42 | 38.55 | 0 | 0 | 1021 | 13.93 | 1.34 | -0.095 | 8.25 | 60.94 | WCMC |
| Et_Nec | Nechisar | Ethiopia | 6.00 | 37.90 | 0 | 0 | 1137 | 18.05 | 0.66 | 0.050 | 9.50 | 64.58 | WCMC; APAAT |
| Et_Sha | AbijattaShalla | Ethiopia | 7.55 | 38.53 | 1 | 4 | 1075 | 17.20 | 0.75 | 0.088 | 9.50 | 66.78 | WCMC |
| Ga_Fab | ForetAbeilles | Gabon | -0.33 | 11.75 | 1 | 4 | 1681 | 23.90 | 1.08 | 0.015 | 9.00 | 38.36 | Gautier-Hion *et al.* 1999 |
| Ga_Lsa | Lekedi | Gabon | -1.78 | 13.02 | 1 |  | 1817 | 23.05 | 0.96 | 0.128 | 8.75 | 40.23 | Coad *et al.* 2010 |
| Ga_Maa | Makande | Gabon | 0.67 | 11.90 | 1 | 4 | 1594 | 24.20 | 0.96 | 0.008 | 9.25 | 41.03 | Fleury & Gautier-Hion 1999; Gautier-Hion *et al.* 1999; Brugiere *et al.* 2002 |
| Ga_May | Mayumba | Gabon | -3.82 | 10.95 | 1 | 3 | 1441 | 24.00 | 1.64 | -0.120 | 8.00 | 30.55 | APAAT & Maisels, *et al.* 2007; Christy *et al.* 2008 |
| Ga_Mib | MIboundji | Gabon | -1.67 | 12.02 | 1 |  | 1896 | 22.30 | 1.11 | 0.138 | 9.00 | 39.86 | Blom *et al.* 1992 |
| Ga_Min | Minkebe | Gabon | 1.72 | 13.00 | 1 | 4 | 1640 | 23.40 | 0.80 | 0.120 | 11.25 | 51.62 | Gautier-Hion & Brugière 2005 |
| Ga_Mwa | Mwagne | Gabon | 0.47 | 13.80 | 1 | 4 | 1572 | 23.25 | 0.83 | 0.123 | 10.25 | 49.51 | Christy *et al.* 2008 |
| Ga_PBa | PlatBateke | Gabon | -2.21 | 14.03 | 1 | 4 | 1941 | 23.23 | 0.81 | 0.178 | 9.00 | 41.20 | Christy *et al.* 2008 |
| Ga_Pon | Pongara | Gabon | 0.15 | 9.62 | 1 | 3 | 2382 | 26.15 | 1.13 | 0.090 | 9.00 | 24.09 | Christy *et al.* 2008 |
| GA_Ptl | PetitLoango | Gabon | -2.27 | 9.62 | 1 | 3 | 1819 | 26.00 | 1.56 | -0.060 | 8.00 | 22.76 | WCM & APAAT & Christy *et al.* 2008 |
| Gh_Abe | AjenjuaBepo | Ghana | 6.37 | -1.03 | 1 | 1 | 1474 | 26.20 | 1.00 | -0.110 | 10.25 | 39.80 | Aalangdong 2009 |
| Gh_BF | Boab-Fiema | Ghana | 7.72 | -1.70 | 1 | 1 | 1366 | 26.30 | 1.22 | -0.173 | 8.75 | 33.20 | Teichroeb *et al.* 2003 |
| Gh_Dig | Digya | Ghana | 7.43 | -0.22 | 1 | 3 | 1415 | 27.20 | 1.26 | -0.203 | 8.50 | 29.03 | WCMC |
| Gh_Mol | Mole | Ghana | 9.65 | -1.76 | 0 | 0 | 1095 | 27.23 | 1.59 | -0.368 | 7.00 | 22.98 | WCMC |
| Gu_Bad | Badiar | Guinea | 12.58 | -13.29 | 0 | 0 | 1282 | 27.23 | 2.28 | -0.430 | 5.25 | 6.35 | APAAT |
| Gu_Csa | ChutesSaala | Guinea | 10.32 | -12.52 | 1 |  | 1967 | 24.40 | 1.43 | -0.113 | 7.25 | 21.91 | APAAT |
| Gu_Itr | IlesTristao | Guinea | 10.95 | -14.95 | 1 | 2 | 2495 | 26.93 | 1.04 | -0.263 | 5.33 | 4.92 | APAAT |
| Ke_Abe | Aberdare | Kenya | -0.41 | 36.74 | 1 | 1 | 1050 | 15.60 | 0.90 | 0.145 | 12.00 | 77.39 | Williams 1967; Lambrechts *et al.* 2003; WCMC |
| Ke_Amb | Amboseli | Kenya | -2.64 | 37.25 | 1 | 1 | 885 | 21.10 | 1.51 | -0.240 | 7.50 | 42.53 | Williams 1967; APAAT; WCMC |
| Ke_Ged | Gede | Kenya | -3.30 | 40.02 | 1 | 1 | 973 | 25.20 | 1.39 | -0.315 | 9.00 | 38.00 | Williams 1967 |
| Ke_Kak | Kakamega | Kenya | 0.32 | 34.87 | 1 | 3 | 1541 | 18.60 | 0.81 | 0.323 | 12.00 | 67.86 | Cords 2012 |
| Ke_Kis | Kisite | Kenya | -4.71 | 39.36 | 1 | 1 | 1038 | 25.40 | 1.58 | -0.280 | 10.00 | 38.26 | Moreno-Black & Maples 1977 |
| Ke_Mar | Marsabit | Kenya | 2.36 | 37.96 | 1 | 1 | 825 | 25.75 | 0.97 | -0.498 | 6.00 | 31.43 | Williams 1967 |
| Ke_Mat | MathewsSamburu | Kenya | 1.26 | 37.28 | 1 | 1 | 875 | 21.58 | 0.77 | -0.298 | 6.75 | 46.44 | Mwenja 2007; de Jong & Butynski 2010 |
| Ke_Mer | Meru | Kenya | 0.10 | 38.22 | 1 | 1 | 950 | 23.90 | 0.84 | -0.323 | 6.50 | 38.85 | Williams 1967; APAAT |
| Ke_MM | Masai-Mara | Kenya | -1.50 | 35.11 | 1 | 2 | 1175 | 19.38 | 0.80 | 0.143 | 10.75 | 65.23 | Williams 1967; APAAT |
| Ke_TR | TanaRiver | Kenya | -1.89 | 40.12 | 1 | 1 | 803 | 26.53 | 1.18 | -0.493 | 6.75 | 30.41 | Karere *et al.* 2004; APAAT |
| Ke_TsE | TsavoEast | Kenya | -2.84 | 38.71 | 1 | 1 | 778 | 24.43 | 1.56 | -0.453 | 5.00 | 25.96 | Williams 1967; APAAT |
| Li_Cmo | CapMount | Liberia | 6.81 | -11.37 | 1 | 4 | 3308 | 25.73 | 0.95 | 0.160 | 9.00 | 14.08 | APAAT |
| Li_Nim | Nimba | Liberia | 7.53 | -8.47 | 1 |  | 2007 | 24.93 | 0.73 | 0.060 | 9.75 | 38.27 | WCMC |
| Li_Nlo | Nlorma | Liberia | 8.03 | -9.74 | 1 | 3 | 2367 | 24.33 | 0.79 | 0.193 | 10.25 | 37.02 | Barrie *et al.* 2007 |
| Ma_AnM | Ansongo-Menaka | Mali | 15.44 | 1.56 | 0 | 0 | 321 | 29.18 | 3.69 | -0.853 | 2.00 | .00 | APAAT |
| Ma_Bad | Badinko | Mali | 13.50 | -9.42 | 0 | 0 | 896 | 27.90 | 3.00 | -0.548 | 4.50 | .00 | APAAT |
| Ma_Baf | Bafing | Mali | 12.62 | -10.53 | 0 | 0 | 999 | 27.80 | 2.59 | -0.508 | 5.00 | 4.16 | WCMC; Kormos *et al.* 2003 |
| Ma_Cba | CliffsBandiagara | Mali | 14.41 | -3.40 | 0 | 0 | 512 | 27.28 | 3.15 | -0.728 | 4.00 | .00 | APAAT |
| Ma_Gou | Gourma | Mali | 15.70 | -2.78 | 0 | 0 | 334 | 28.90 | 3.78 | -0.843 | 2.75 | .00 | APAAT |
| Mo_Baz | Bazaruto | Mozambique | -21.75 | 35.43 | 1 | 1 | 804 | 24.40 | 2.44 | -0.460 | 5.00 | 16.09 | WCMC; APAAT |
| Mo_Gil | Gile | Mozambique | -16.53 | 38.44 | 1 | 1 | 1228 | 25.15 | 2.36 | -0.243 | 7.00 | 18.75 | APAAT |
| Mo_Gor | Gorongosa | Mozambique | -18.82 | 34.51 | 1 | 1 | 1084 | 25.03 | 2.64 | -0.343 | 6.00 | 13.46 | APAAT |
| Mo_Map | Maputo | Mozambique | -18.77 | 35.95 | 1 | 1 | 1049 | 25.65 | 2.30 | -0.340 | 7.00 | 19.58 | APAAT |
| Mo_Nia | Niassa | Mozambique | -12.24 | 36.98 | 0 | 0 | 1279 | 23.60 | 1.66 | -0.295 | 6.00 | 24.58 | APAAT |
| Mo_Pom | Pomene | Mozambique | -23.00 | 35.55 | 1 | 1 | 935 | 23.70 | 2.34 | -0.315 | 7.00 | 25.07 | APAAT; WCMC |
| Mo_Zin | Zinave | Mozambique | -21.59 | 33.54 | 1 | 1 | 632 | 24.23 | 2.02 | -0.598 | 4.75 | 20.59 | APAAT |
| Mu_Cha | ChatTboul | Mauritania | 16.55 | -16.40 | 0 | 0 | 261 | 26.00 | 2.28 | -0.865 | 2.00 | 7.16 | WCMC; RAMSAR accessed 2003-2006 |
| Mw_Chi | Chilwa | Malawi | -15.27 | 35.68 | 1 | 1 | 1410 | 23.20 | 2.41 | -0.143 | 7.00 | 20.84 | APAAT |
| Mw_Kas | Kasungu | Malawi | -12.94 | 33.14 | 1 | 1 | 938 | 21.10 | 2.54 | -0.365 | 6.00 | 24.06 | APAAT; WCMC; Garine-Wichatitsky *et al.* 2001 |
| Mw_Maj | Majete | Malawi | -15.93 | 34.59 | 1 | 1 | 953 | 25.08 | 2.61 | -0.428 | 5.75 | 13.56 | APAAT |
| Mw_Mal | Malawi | Malawi | -14.06 | 34.87 | 1 | 1 | 963 | 22.95 | 2.44 | -0.413 | 5.50 | 18.84 | APAAT; WCMC |
| Mw_Nyi | Nyika | Malawi | -10.55 | 33.83 | 1 | 1 | 1172 | 20.68 | 2.14 | -0.190 | 7.00 | 31.51 | APAAT; WCMC |
| Na_AAi | Ai-Ais | Namibia | -27.91 | 17.15 | 0 | 0 | 84 | 17.20 | 3.39 | -0.868 | 0.00 | 8.85 | Griffin & Griffin 1998; APAAT; WCMC |
| Na_DVi | DaanViljoen | Namibia | -22.53 | 16.96 | 0 | 0 | 335 | 18.20 | 3.71 | -0.680 | 3.25 | 12.76 | Griffin & Griffin 1998; APAAT; WCMC |
| Na_Eto | Etosha | Namibia | -19.00 | 15.75 | 0 | 0 | 392 | 22.20 | 3.46 | -0.720 | 4.00 | 8.49 | APAAT; WCMC |
| Na_Har | Hardap | Namibia | -24.50 | 17.77 | 0 | 0 | 211 | 20.10 | 4.71 | -0.825 | 2.25 | .00 | APAAT |
| Na_Kha | Khaudum | Namibia | -18.78 | 20.76 | 0 | 0 | 560 | 21.60 | 3.92 | -0.613 | 5.00 | 7.01 | APAAT |
| Na_NNa | Namib-Naukluft | Namibia | -24.55 | 15.33 | 0 | 0 | 159 | 15.45 | 2.40 | -0.813 | 2.25 | 29.66 | APAAT; WCMC |
| Na_Ske | Skeleton-Coast | Namibia | -19.28 | 12.80 | 0 | 0 | 152 | 15.33 | 1.77 | -0.800 | 3.00 | 39.29 | APAAT; WCMC |
| Na_Wat | Waterberg | Namibia | -20.44 | 17.25 | 0 | 0 | 447 | 19.20 | 3.77 | -0.605 | 4.00 | 11.99 | APAAT; WCMC |
| Ng_Add | Addax | Niger | 19.31 | 8.37 | 0 | 0 | 51 | 25.15 | 5.42 | -0.970 | 0.00 | .00 | APAAT |
| Ng_AiT | AirTenere | Niger | 18.83 | 9.47 | 0 | 0 | 62 | 25.35 | 5.46 | -0.968 | 0.00 | .00 | APAAT |
| Ng_Gad | Gadabedji | Niger | 15.14 | 7.15 | 0 | 0 | 434 | 27.88 | 3.60 | -0.783 | 3.00 | .00 | APAAT |
| Ni_Afi | AfiMountain | Nigeria | 6.33 | 8.95 | 1 | 4 | 2030 | 27.48 | 1.13 | -0.080 | 8.00 | 21.01 | Bassey 2012; http://www.westerngorilla.org/Afi.htm |
| Ni_Bat | Baturiya | Nigeria | 12.52 | 10.42 | 0 | 0 | 544 | 26.90 | 3.19 | -0.720 | 3.75 | .00 | APAAT |
| Ni_Dag | Dagida | Nigeria | 9.66 | 5.47 | 0 | 0 | 1144 | 27.38 | 1.76 | -0.378 | 6.75 | 19.19 | APAAT |
| Ni_GaG | Gashaka-Gumti | Nigeria | 7.49 | 7.65 | 1 |  | 1333 | 27.20 | 1.50 | -0.263 | 7.00 | 22.06 | WCMC; APAAT |
| Ni_Gba | Gbanraun | Nigeria | 4.92 | 6.00 | 1 | 3 | 2898 | 26.75 | 0.99 | 0.155 | 10.00 | 20.84 | Grubb & Powell 1999; Werre 2000 |
| Ni_Kai | Kainji | Nigeria | 10.04 | 4.09 | 0 | 0 | 1124 | 27.00 | 1.97 | -0.388 | 6.75 | 17.83 | APAAT; WCMC |
| Ni_Kam | Kamuku | Nigeria | 10.80 | 6.30 | 0 | 0 | 1080 | 25.35 | 1.89 | -0.383 | 5.75 | 19.53 | APAAT; WCMC |
| Ni_Oko | Okomu | Nigeria | 6.32 | 5.24 | 1 | 3 | 1875 | 26.68 | 1.21 | -0.050 | 8.50 | 26.06 | APAAT |
| Ni_Yan | Yankari | Nigeria | 9.82 | 10.50 | 0 | 0 | 1024 | 26.60 | 2.19 | -0.460 | 5.50 | 12.72 | Kadiri 2014 |
| Rw_Aka | Akagera | Rwanda | -1.65 | 30.69 | 1 | 2 | 856 | 19.70 | 0.30 | -0.105 | 9.00 | 65.45 | APAAT; WCMC |
| Rw_Gis | Gishwati | Rwanda | -1.75 | 29.43 | 1 | 4 | 1196 | 19.85 | 0.32 | 0.150 | 10.50 | 68.48 | APAAT |
| SA_Aug | Augrabies | South Africa | -28.52 | 20.14 | 0 | 0 | 124 | 19.75 | 5.50 | -0.868 | 0.00 | .00 | APAAT; WCMC |
| SA_BAl | B-Alberts | South Africa | -24.62 | 27.35 | 0 | 0 | 587 | 22.00 | 4.38 | -0.585 | 5.75 | 3.65 | APAAT |
| SA_Bon | Bontebok | South Africa | -34.07 | 20.47 | 0 | 0 | 451 | 15.60 | 3.40 | -0.268 | 9.00 | 43.79 | APAAT |
| SA_CaP | Cape-Peninsula | South Africa | -34.14 | 18.41 | 0 | 0 | 649 | 17.30 | 3.47 | -0.150 | 7.00 | 32.30 | APAAT |
| SA_CVi | Cape Vidal | South Africa | -28.08 | 32.55 | 1 | 1 | 981 | 21.90 | 2.65 | -0.120 | 10.00 | 36.97 | APAAT |
| SA_Doo | Doorndraai | South Africa | -24.31 | 28.76 | 1 | 1 | 605 | 18.58 | 3.97 | -0.443 | 7.00 | 20.59 | van der Zee & Skinner 1977; APAAT; WCMC |
| SA_Erf | Erfenis | South Africa | -28.50 | 26.80 | 0 | 0 | 584 | 16.10 | 5.06 | -0.338 | 7.00 | 15.70 | APAAT; WCMC |
| SA_Fai | Fairbreeze | South Africa | -27.87 | 30.21 | 1 | 1 | 778 | 15.88 | 3.94 | -0.148 | 8.00 | 31.29 | NBA 2011 |
| SA_Gab | Gamkaberg | South Africa | -33.72 | 21.90 | 0 | 0 | 278 | 16.13 | 3.97 | -0.550 | 1.50 | 11.42 | APAAT |
| SA_GiC | GiantsCastle | South Africa | -29.22 | 29.49 | 0 | 0 | 861 | 13.73 | 4.18 | 0.028 | 8.25 | 35.18 | WCMC |
| SA_Goe | Goegap | South Africa | -29.66 | 18.02 | 0 | 0 | 151 | 17.88 | 4.51 | -0.683 | 0.00 | .00 | APAAT |
| SA_Gou | Goukamma | South Africa | -34.05 | 22.90 | 0 | 0 | 706 | 13.75 | 3.17 | 0.050 | 12.00 | 60.35 | APAAT |
| SA_Hla | Hlane | South Africa | -26.25 | 31.88 | 1 | 1 | 699 | 22.40 | 3.09 | -0.470 | 6.50 | 19.14 | WCMC |
| SA_HMe | H-Merensky | South Africa | -23.69 | 30.67 | 1 | 1 | 1011 | 21.75 | 2.82 | -0.273 | 6.50 | 21.69 | APAAT; WCMC |
| SA_Ita | Itala | South Africa | -27.51 | 31.29 | 1 | 1 | 777 | 19.50 | 2.93 | -0.268 | 7.50 | 31.63 | APAAT; WCMC |
| SA_Kar | Karoo | South Africa | -32.27 | 22.29 | 0 | 0 | 217 | 16.93 | 4.47 | -0.693 | 0.75 | .87 | APAAT; WCMC |
| SA_KGe | Kalahari Gemsbok | South Africa | -25.62 | 20.43 | 0 | 0 | 209 | 19.90 | 5.37 | -0.820 | 1.50 | .00 | APAAT; WCMC |
| SA_Laj | Lajuma | South Africa | -23.03 | 29.43 | 1 | 1 | 571 | 19.88 | 3.59 | -0.508 | 5.25 | 16.14 | Emerson, *et al.* 2011 |
| SA_Mad | Madikwe | South Africa | -24.76 | 26.30 | 0 | 0 | 583 | 20.23 | 4.65 | -0.523 | 7.00 | 9.16 | *http://www.parksnorthwest.co.za/madikwe/madikwe_animals.html;* APAAT; WCMC |
| SA_Mat | Matjiesriver | South Africa | -32.49 | 19.41 | 0 | 0 | 302 | 16.63 | 4.59 | -0.420 | 3.75 | 12.48 | APAAT; WCMC |
| SA_MMa | MalaMala | South Africa | -24.84 | 31.53 | 0 | 0 | 804 | 21.80 | 3.43 | -0.385 | 6.25 | 15.62 | *http://www.malamala.com/mamlist.htm; WCMC* |
| SA_Ori | OribiGorge | South Africa | -30.71 | 30.27 | 1 | 1 | 1008 | 18.37 | 2.52 | 0.057 | 10.67 | 49.70 | APAAT; WCMC |
| SA_Ovi | Oviston | South Africa | -30.63 | 25.57 | 0 | 0 | 424 | 15.13 | 5.30 | -0.425 | 7.00 | 16.07 | APAAT; WCMC |
| SA_Sui | Suikerbosrand | South Africa | -26.51 | 28.25 | 0 | 0 | 690 | 15.90 | 4.29 | -0.270 | 7.00 | 23.80 | APAAT; WCMC |
| SA_TBa | T-Baines | South Africa | -33.39 | 26.49 | 0 | 0 | 581 | 17.33 | 2.69 | -0.227 | 9.67 | 48.80 | APAAT; WCMC |
| SA_Tsi | Tsitsikamma | South Africa | -34.04 | 23.99 | 0 | 0 | 830 | 13.30 | 2.98 | 0.190 | 12.00 | 63.58 | APAAT; WCMC; http://www.parks-sa.co.za/parks/Tsitsikamma/default.html#fauna |
| SA_Vaa | Vaalbos | South Africa | -28.59 | 24.34 | 0 | 0 | 412 | 18.18 | 5.54 | -0.578 | 5.25 | .00 | APAAT; WCMC |
| SA_Zeb | Mt-Zebra | South Africa | -32.20 | 25.40 | 0 | 0 | 343 | 15.00 | 4.57 | -0.538 | 5.75 | 19.72 | APAAT; WCMC; http://www.parks-sa.co.za/parks/Tsitsikamma/default.html#fauna |
| Se_BsC | BasseCasamance | Senegal | 12.40 | -16.60 | 1 | 2 | 1309 | 26.00 | 1.34 | -0.475 | 5.00 | 16.96 | APAAT; WCMC |
| Se_FeS | FerloSud | Senegal | 14.85 | -14.03 | 0 | 0 | 625 | 28.55 | 2.62 | -0.693 | 4.00 | 1.98 | APAAT |
| Se_Fon | Fongoli | Senegal | 12.65 | -11.78 | 0 | 0 | 1334 | 27.80 | 2.47 | -0.408 | 5.50 | 3.56 | Pers. comm. J. Pruetz; http://mahale.web.infoseek.co.jp/PAN/11_2/112_04.html |
| Se_Sal | Saloum | Senegal | 13.97 | -16.59 | 0 | 0 | 754 | 26.73 | 1.47 | -0.637 | 3.67 | 15.78 | APAAT |
| SL_Lom | Loma-Mts | Sierra Leone | 9.16 | -11.11 | 1 | 2 | 2348 | 25.20 | 1.10 | 0.063 | 8.75 | 25.58 | *http://www.daco-sl.org/encyclopedia2004/2_data/2_1/2_1d_env/Biodiversity_national_rpt03.pdf accessed 2004 - no longer available; APAAT.* |
| SL_WAr | West-Area | Sierra Leone | 8.34 | -13.17 | 1 | 2 | 2516 | 26.60 | 1.00 | -0.060 | 8.00 | 17.16 | *http://www.daco-sl.org/encyclopedia2004/2_data/2_1/2_1d_env/Biodiversity_national_rpt03.pdf accessed 2004 - no longer available; APAAT* |
| SP_STo | SaoTome | Sao Tome and Principe | 0.17 | 6.67 | 1 | 1 | 1507 | 25.10 | 0.86 | -0.020 | 9.00 | 40.43 | Glenn *et al.* 2002 |
| Ta_Kat | Katavi | Tanzania | -6.98 | 31.32 | 1 | 1 | 979 | 29.15 | 1.13 | -0.493 | 6.00 | 20.82 | APAAT; WCMC |
| Ta_Mah | Mahale | Tanzania | -6.23 | 29.93 | 1 | 2 | 1076 | 21.83 | 0.84 | -0.175 | 7.25 | 45.63 | Nishida & Hosaka 1996 |
| Ta_Man | Manyara | Tanzania | -3.49 | 35.81 | 1 | 1 | 868 | 19.85 | 1.58 | -0.208 | 7.00 | 43.41 | APAAT; Tanzania National Parks 1979 |
| Ta_Mik | Mikumi | Tanzania | -7.34 | 37.25 | 1 | 1 | 1312 | 23.55 | 1.74 | -0.165 | 7.00 | 28.46 | APAAT; WCMC |
| Ta_Mko | Mkomazi | Tanzania | -4.17 | 38.27 | 1 | 1 | 807 | 23.38 | 1.67 | -0.368 | 7.25 | 34.75 | Eltringham *et al.* 1999; APAAT |
| Ta_Nda | NewDabaga | Tanzania | -8.13 | 35.92 | 1 | 1 | 1063 | 19.43 | 1.59 | -0.210 | 6.50 | 39.60 | Marshall *et al.* 2005 |
| Ta_Rli | Rungwe-Livingstone | Tanzania | -9.15 | 33.76 | 1 | 1 | 1544 | 17.48 | 1.98 | 0.065 | 8.00 | 40.99 | Davenport, *et al.* 2014 |
| Ta_Rua | Ruaha | Tanzania | -7.50 | 34.64 | 1 | 1 | 717 | 20.80 | 1.58 | -0.393 | 5.50 | 36.26 | APAAT; WCMC |
| Ta_Rub | Rubondo | Tanzania | -2.33 | 31.85 | 0 | 0 | 1070 | 21.90 | 0.41 | -0.075 | 8.75 | 56.43 | APAAT; WCMC |
| Ta_Saa | Saadani | Tanzania | -6.14 | 38.74 | 1 | 1 | 1110 | 25.43 | 1.54 | -0.283 | 8.50 | 32.82 | APAAT; WCMC |
| Ta_Sel | Selous | Tanzania | -8.85 | 37.45 | 1 | 1 | 1301 | 24.25 | 1.68 | -0.248 | 7.00 | 26.63 | Davenport *et al.* 2014 |
| TG_Rga | RiverGambia | The Gambia | 13.64 | -15.00 | 0 | 0 | 818 | 27.68 | 2.03 | -0.598 | 4.75 | 10.93 | APAAT |
| To_Fli | FosseLions | Togo | 10.76 | 0.21 | 0 | 0 | 964 | 25.03 | 1.60 | -0.415 | 6.00 | 25.29 | Campbell *et al.* 2008 |
| To_Fma | Fazao-Malfakassa | Togo | 8.77 | 0.82 | 1 | 1 | 1569 | 25.45 | 1.55 | -0.148 | 7.50 | 24.78 | Campbell *et al.* 2008 |
| Ug_Bud | Budongo | Uganda | 1.73 | 31.55 | 1 | 2 | 1188 | 23.80 | 0.75 | -0.063 | 10.50 | 52.97 | Tweheyo & Obua 2001 |
| Ug_Cho | Chobe | Uganda | 2.25 | 32.15 | 1 |  | 1239 | 24.45 | 0.87 | -0.075 | 9.00 | 44.77 | Oates 1977 |
| Ug_Kar | Kampala | Uganda | 0.17 | 32.53 | 1 | 1 | 1463 | 21.58 | 0.59 | 0.223 | 12.00 | 64.01 | Baranga 2004 |
| Ug_Kib | Kibale | Uganda | 0.37 | 30.42 | 1 | 3 | 1200 | 21.00 | 0.46 | 0.078 | 11.25 | 65.88 | Chapman *et al.* 2002; APAAT |
| Ug_Kid | Kidepo | Uganda | 3.85 | 33.78 | 1 | 1 | 837 | 23.90 | 1.15 | -0.323 | 8.00 | 41.90 | APAAT |
| Ug_Tor | Tororo | Uganda | 0.73 | 43.18 | 1 | 3 | 548 | 27.10 | 1.13 | -0.660 | 4.33 | 23.49 | Decker 1995 |
| Za_Ban | Bangweulu | Zambia | -11.89 | 30.12 | 1 | 1 | 1255 | 20.63 | 2.22 | -0.260 | 6.00 | 25.29 | APAAT |
| Za_BlL | BlueLagoon | Zambia | -15.49 | 27.41 | 0 | 0 | 811 | 21.08 | 3.06 | -0.448 | 5.00 | 16.03 | APAAT |
| Za_Kaf | Kafue | Zambia | -15.38 | 25.64 | 0 | 0 | 836 | 20.25 | 3.33 | -0.415 | 5.00 | 14.89 | APAAT |
| Za_Liu | Liuwa | Zambia | -14.49 | 22.57 | 0 | 0 | 971 | 21.43 | 2.61 | -0.355 | 5.25 | 19.99 | APAAT |
| Za_LuP | LusengaPlain | Zambia | -9.40 | 29.22 | 1 | 1 | 1220 | 22.80 | 1.56 | -0.198 | 7.00 | 32.74 | APAAT |
| Za_Lza | LowerZambezi | Zambia | -15.44 | 29.68 | 1 | 1 | 752 | 22.20 | 3.16 | -0.498 | 5.00 | 12.88 | APAAT |
| Za_Slu | SouthLuangwa | Zambia | -13.09 | 31.53 | 1 | 1 | 965 | 23.25 | 2.64 | -0.430 | 5.00 | 14.25 | APAAT |
| Zi_Chi | Chizarira | Zimbabwe | -17.77 | 27.93 | 0 | 0 | 721 | 21.68 | 3.36 | -0.500 | 5.00 | 12.17 | APAAT |
| Zi_Hwa | Hwange | Zimbabwe | -19.19 | 26.62 | 0 | 0 | 692 | 20.78 | 3.39 | -0.500 | 5.00 | 14.05 | APAAT |

**Table SV** Further details on the selection process conducted to find the best fit equations for time budget and diet using R version 3.3.1

Time budget equations were obtained by identifying the best fit general mixed model using the lme4 package in R. We also used the MuMIn package to help with model selection.

**Leaf:**

Global model call: lmer(formula = Leaf ~ BM_afam + P_ann + l10_Tann + Tmosd + Moimo + P.2T + (1 | Site) + (1 | Species), data = guendiet, REML = FALSE)

---

Model selection table

| **Model** | **(Int)** | **bm_afm** | **Mom** | **P_ann** | **P.2T** | **Tms** | **df** | **logLik** | **AICc** | **delta** | **weight** |
| --- | --- | --- | --- | --- | --- | --- | --- | --- | --- | --- | --- |
| 5 | 22.05 |  | -36.68 |  |  |  | 5 | -103.798 | 220.3 | 0 | 0.373 |
| 13 | 3.374 |  | -44.02 | 0.01008 |  |  | 6 | -102.624 | 221.2 | 0.92 | 0.235 |
| **6** | -2.353 | 5.74 | -36.91 |  |  |  | 6 | -103.133 | 222.3 | 1.94 | 0.141 |
| 21 | 30.64 |  | -34.13 |  | -0.864 |  | 6 | -103.637 | 223.3 | 2.95 | 0.085 |
| 14 | -20.27 | 5.479 | -44.28 | 0.01026 |  |  | 7 | -101.861 | 223.3 | 3 | 0.083 |
| 37 | 20.04 |  | -31.67 |  |  | 1.787 | 6 | -103.68 | 223.4 | 3.04 | 0.082 |

Models ranked by AICc(x)

Random terms (all models): ‘1 | Site’, ‘1 | Species’

The top model only included Moisture as an explanatory variable. The second model includes rainfall, but rainfall and moisture are highly dependent on each other and we preferred not to have those two variables together in one equation. Therefore, we opted for the third equation which makes biological sense in that percentage leaf in the diet is known to be related to body size and forest productivity is often related to moisture. Leaves are not considered the preferred diets of guenons.

We then tested the performance of this model with just the predictor variables in it against a null model with only the random factors and 1:

anova(diet.null,diet.best)

Data: guendiet

Models:

diet.null: Leaf ~ 1 + (1 | Site) + (1 | Species)

diet.best: Leaf ~ BM_afam + Moimo + (1 | Site) + (1 | Species)

| Mode | Df | AIC | AICc | BIC | LogLik | deviance | Chisq | df | P |
| --- | --- | --- | --- | --- | --- | --- | --- | --- | --- |
| Null | 4 | 222.26 | 224.00 | 227.59 | -107.13 | 214.26 |  |  |  |
| Leaf | 6 | 218.27 | 222.27 | 226.26 | -103.13 | 206.27 | 7.9901 | 2 | 0.018 |

Linear mixed model fit by maximum likelihood ['lmerMod']

Formula: Leaf ~ BM_afam + Moimo + (1 | Site) + (1 | Species)

Data: guendiet

| AIC | BIC | logLik | deviance | df.resid |
| --- | --- | --- | --- | --- |
| 218.3 | 226.3 | -103.1 | 206.3 | 22 |

Scaled residuals:

| Min | 1Q | Median | 3Q | Max |
| --- | --- | --- | --- | --- |
| -2.09932 | -0.52061 | 0.03953 | 0.44307 | 1.96157 |

Random effects:

| Groups | Name | Variance | Std.Dev. |
| --- | --- | --- | --- |
| Site | (Intercept) | 0 | 0 |
| Species | (Intercept) | 128.69 | 11.344 |
| Residual | | 46.53 | 6.821 |

Number of obs: 28, groups: Site, 19; Species, 10

**Fixed effects:**

|  | Estimate | Std. Error | t value |
| --- | --- | --- | --- |
| **(Intercept)** | -2.353 | 20.893 | -0.113 |
| **BM_afam** | 5.74 | 4.84 | 1.186 |
| **Moimo** | -36.911 | 11.935 | -3.093 |

Correlation of Fixed Effects:

|  | (Intr) | bm_afm |
| --- | --- | --- |
| BM_afam | -0.981 |  |
| Moimo | 0.019 | -0.053 |

**Moving time Model fit ranked according to AICc**

Global model call: lmer(formula = move ~ BM_afam + Leaf + log10_Nsp + grpsz_st +

l10_Tann + P_ann + Tmosd + P>2T + (1 | site) + (1 |

species), data = guenons, REML = FALSE)

---

Model selection table

|  | (Int) | BM_afm | grpsize | leaf | p>2T | df | logLik | AICc | delta | weight |
| --- | --- | --- | --- | --- | --- | --- | --- | --- | --- | --- |
| 2 | 0 | -0.5812 |  |  |  | 5 | -46.414 | 109.5 | 0 | 0.437 |
| 1 | 0 |  |  |  |  | 4 | -49.103 | 110.2 | 0.71 | 0.306 |
| 3 | 0 |  | -0.3883 |  |  | 5 | -47.708 | 112.1 | 2.59 | 0.12 |
| 34 | 0 | -0.6249 |  |  | -0.2527 | 6 | -45.677 | 113.9 | 4.36 | 0.049 |
| 4 | 0 | -0.5084 | -0.2389 |  |  | 6 | -45.804 | 114.1 | 4.61 | 0.044 |

Models ranked by AICc(x)

Random terms (all models):

‘1 | site’, ‘1 | species’

**Moving time Model fit ranked according to AIC**

Global model call: lmer(formula = move ~ BM_afam + Leaf + log10_Nspp + grpsize +

Log10_Tann + Pann + Tmosd + P>2t + (1 | site) + (1 | species), data = guenons, REML = FALSE)

---

Model selection table

|  | (Int) | BM_afm | grp_st | log10Nsp | l10Tnn | lfm | p2t | Pann | tmosd | df | logLik | AIC | delta | weight |
| --- | --- | --- | --- | --- | --- | --- | --- | --- | --- | --- | --- | --- | --- | --- |
| 215 | 0 |  | -1.124 | 0.4837 |  | -0.5649 |  | -0.555 | 0.6821 | 9 | -32.877 | 83.8 | 0 | 0.665 |
| 223 | 0 |  | -1.093 | 0.9132 | 0.5866 | -0.7097 |  | -0.666 | 0.9651 | 10 | -32.563 | 85.1 | 1.37 | 0.335 |
| 2 | 0 | -0.5812 |  |  |  |  |  |  |  | 5 | -46.414 | 102.8 | 19.07 | 0 |
| **34** | **0** | **-0.6249** |  |  |  |  | **-0.2527** |  |  | **6** | **-45.677** | **103.4** | **19.6** | **0** |
| 116 | 0 | -0.5945 | -0.3629 |  |  | -0.273 | -0.3909 | -0.365 |  | 9 | -42.709 | 103.4 | 19.66 | 0 |
| 36 | 0 | -0.5453 | -0.2847 |  |  |  | -0.2942 |  |  | 7 | -44.721 | 103.4 | 19.69 | 0 |

Models ranked by AIC(x)

Random terms (all models):

‘1 | site’, ‘1 | species’

**Moving time Model selected against null model anova output**

> move.null = lmer(move ~ 1 + (1|site) + (1|species), data=guenons, REML=FALSE)

> anova(move.null, move.best)

Data: guenons

Models:

move.null: move ~ 1 + (1 | site) + (1 | species)

move.best: move ~ BM_afam + P>2T + (1 | site) + (1 | species)

| Model | Df | AIC | AICc | BIC | LogLik | deviance | Chisq | df | P |
| --- | --- | --- | --- | --- | --- | --- | --- | --- | --- |
| Null | 4 | 106.21 | 110.21 | 109.04 | -49.10 | 98.21 |  |  |  |
| Move | 6 | 103.36 | 113.85 | 107.60 | -45.68 | 91.36 | 6.85 | 2 | 0.033 |

**> summary(move.best)**

Linear mixed model fit by maximum likelihood ['lmerMod']

Formula: move ~ BM_afam + P>2T + (1 | site) + (1 | species)

Data: guenons

| AIC | BIC | logLik | deviance | df.resid |
| --- | --- | --- | --- | --- |
| 103.4 | 107.6 | -45.7 | 91.4 | 9 |

Scaled residuals:

| Min | 1Q | Median | 3Q | Max |
| --- | --- | --- | --- | --- |
| -2.09219 | -0.46868 | 0.09819 | 0.60715 | 1.95925 |

Random effects:

| Groups | Name | Variance | Std.Dev. |
| --- | --- | --- | --- |
| site | (Intercept) | 0 | 0 |
| species | (Intercept) | 0 | 0 |
| Residual | | 25.85 | 5.085 |

Number of obs: 15, groups: site, 10; species, 6

**Fixed effects**

|  | Estimate | Std. Error | t value |
| --- | --- | --- | --- |
| **(Intercept)** | 67.236 | 19.353 | 3.474 |
| **BM_afam** | -5.032 | 1.636 | -3.076 |
| **P>2T** | -1.939 | 1.559 | -1.244 |

Correlation of Fixed Effects:

|  | (Intr) | BM_afm |
| --- | --- | --- |
| BM_afam | -0.55 |  |
| P>2T | -0.915 | 0.173 |

**Feeding time model comparisons based on AICc**

During the selection process we rescaled rainfall to P_ann/1000 (*Pann1000*).

The best fit model based on AICc is the one we used for the time budget model.

The top best fit model based on AIC has too many variables to be reliable and again the best fit model with at most 2 variables in it is the one that came include rainfall and group size.

This is the model we used to determine the time budget equation for feeding time.

Global model call: lmer(formula = feedforage ~ BM_afam + Leaf + log10_Nsp + grpsz_st +

l10_Tann + Pann1000 + Tmosd + P>2T + (1 | site) + (1 | species), data = guenons, REML = FALSE)

---

Model selection table

|  | (Int) | grp_st | log10_Nsp | lfm | p2t_oc | P10 | tms_oc | df | logLik | AICc | delta | weight |
| --- | --- | --- | --- | --- | --- | --- | --- | --- | --- | --- | --- | --- |
| 67 | 0 | 0.507 |  |  |  | 0.495 |  | 6 | -33.924 | 90.3 | 0 | 0.456 |
| 35 | 0 | 0.507 |  |  | 0.486 |  |  | 6 | -34.797 | 92.1 | 1.74 | 0.191 |
| 7 | 0 | 0.507 | 0.321 |  |  |  |  | 6 | -34.896 | 92.3 | 1.94 | 0.173 |
| 131 | 0 | 0.507 |  |  |  |  | -0.256 | 6 | -35.719 | 93.9 | 3.59 | 0.076 |
| 19 | 0 | 0.489 |  | 0.0834 |  |  |  | 6 | -35.839 | 94.2 | 3.83 | 0.067 |
| 3 | 0 | 0.507 |  |  |  |  |  | 5 | -39.356 | 95.4 | 5.03 | 0.037 |

Models ranked by AICc(x)

Random terms (all models):

‘1 | site’, ‘1 | species’

**Feeding time model comparisons based on AIC**

Global model call: lmer(formula = feedforage ~ BM_afam + Leaf + log10_Nsp + grpsz_st + log10_Tann + Pann1000 + Tmosd + P>2T + (1 | site) + (1 | species), data = guenons, REML = FALSE)

---

Model selection table

|  | (Int) | BMafm | Grp_st | log10_Nsp | lfm | p2t | P10 | tms | df | logLik | AIC | delta | weight |
| --- | --- | --- | --- | --- | --- | --- | --- | --- | --- | --- | --- | --- | --- |
| 100 | 0 | -0.092 | 0.507 |  |  | 0.606 | 0.608 |  | 8 | -30.71 | 77.4 | 0 | 0.34 |
| 228 | 0 | -0.103 | 0.507 |  |  | 0.609 | 0.592 | -0.075 | 9 | -30.251 | 78.5 | 1.08 | 0.20 |
| 103 | 0 |  | 0.507 | -0.034 |  | 0.631 | 0.657 |  | 8 | -31.284 | 78.6 | 1.15 | 0.19 |
| 232 | 0 | -0.090 | 0.507 | -0.328 |  | 0.608 | 0.690 | -0.319 | 10 | -29.921 | 79.8 | 2.42 | 0.10 |
| **67** | **0** |  | **0.507** |  |  |  | **0.495** |  | **6** | **-33.924** | **79.8** | **2.43** | **0.10** |
| 116 | 0 | -0.041 | 0.501 |  | 0.027 | 0.617 | 0.626 |  | 9 | -31.14 | 80.3 | 2.86 | 0.08 |

Models ranked by AIC(x)

Random terms (all models):

‘1 | site’, ‘1 | species’

**Feeding time model performance of best fit model against null model**

> anova(feed.null, feed.best)

Data: guenons

Models:

feed.null: feedforage ~ 1 + (1 | site) + (1 | species)

feed.best: feedforage ~ grpsz_st + P_ann + (1 | site) + (1 | species)

| Mode | Df | AIC | AICc | BIC | LogLik | deviance | Chisq | df | P |
| --- | --- | --- | --- | --- | --- | --- | --- | --- | --- |
| Null | 4 | 106.44 | 110.44 | 109.27 | -49.22 | 98.44 |  |  |  |
| Feedforage | 6 | 80.883 | 91.38 | 85.13 | -34.44 | 68.88 | 29.56 | 2 | 3.82e-07 |

> summary(feed.best)

Linear mixed model fit by maximum likelihood ['lmerMod']

Formula: feedforage ~ grpsz_st + P_ann + (1 | site) + (1 | species)

Data: guenons

| AIC | BIC | logLik | deviance | df.resid |
| --- | --- | --- | --- | --- |
| 80.9 | 85.1 | -34.4 | 68.9 | 9 |

Scaled residuals:

| Min | 1Q | Median | 3Q | Max |
| --- | --- | --- | --- | --- |
| -7.18E-07 | -3.26E-07 | 8.94E-08 | 3.03E-07 | 9.17E-07 |

Random effects:

| Groups | Name | Variance | Std.Dev. |
| --- | --- | --- | --- |
| site | (Intercept) | 2.65E+01 | 5.15E+00 |
| species | (Intercept) | 3.14E+01 | 5.61E+00 |
| Residual |  | 6.07E-12 | 2.46E-06 |

Number of obs: 15, groups: site, 10; species, 6

**Fixed effects:**

|  | Estimate | Std. Error | t value |
| --- | --- | --- | --- |
| **(Intercept)** | 1.291e+01 | 8.53E+00 | 2 |
| **grpsz_st** | 3.90E-01 | 1.67E-07 | 2333662 |
| **P_ann** | 9.27E-03 | 4.40E-03 | 2 |

Correlation of Fixed Effects:

|  | Intr | Group size |
| --- | --- | --- |
| grpsz_st | 0.000 |  |
| P_ann | -0.938 | 0.000 |

**Table SVIa: Overview of the robustness analysis showing the name of the amended version (matching the tables shown below) (as expected values, alpha’ was adjusted for multiple tests, n=24, using the Bonferroni method with the Holm’s adjustment, Holm 1979) which gives a p’ of <0.0038 as being considered significant, p’<0.002 is required if we would use the basic Bonferroni method**. The rows representing the equation in which one equation had one slope parameter adjusted by plus or minus 50% of the original slope parameter are highlighted in light grey. As expected, most 50% adjustments lead to a significant difference in predictions but almost all adjustments of 10% have no effect on the predictions of the model. The two exceptions in which p<0.05 are highlighted in dark grey. Only one of these is still significant after the Bonferroni correction. This is the equation for moving time in which the slope value for body mass is decreased by 10% (highlighted in dark grey).

| **Test_nr** | **TB component** | **Variable** | **Slope** | **Change** | **Chi** | ***P*** |
| --- | --- | --- | --- | --- | --- | --- |
| 1 | Feed | Group size N | 0.390 | plus 10% | 0.83 | 0.362 |
| 2 |  |  |  | min 10% | 0.369 | 0.544 |
| 3 |  |  |  | plus 50% | 18.085 | 0.000 |
| 4 |  |  |  | min 50% | 13.287 | 0.000 |
| 5 |  | P_ann | 0.00927 | plus 10% | 2.791 | 0.095 |
| 6 |  |  |  | min 10% | 0.83 | 0.362 |
| 7 |  |  |  | plus 50% | 31.579 | 0.000 |
| 8 |  |  |  | min 50% | 14.417 | 0.000 |
| 9 | Move | BM_afam | -5.032 | plus 10% | 5.905 | 0.015 |
| 10 |  |  |  | min 10% | 8.327 | 0.004 |
| 11 |  |  |  | plus 50% | 50.956 | 0.000 |
| 12 |  |  |  | min 50% | 85.833 | 0.000 |
| 13 |  | P>2T | -1.939 | plus 10% | 0.577 | 0.448 |
| 14 |  |  |  | min 10% | 3.898 | 0.048 |
| 15 |  |  |  | plus 50% | 19.4 | 0.000 |
| 16 |  |  |  | min 50% | 46.711 | 0.000 |
| 17 | Rest | Leaf: BM_afam | 5.740 | plus 10% | 0.83 | 0.362 |
| 18 |  |  |  | min 10% | 0.208 | 0.649 |
| 19 |  |  |  | plus 50% | 15.593 | 0.000 |
| 20 |  |  |  | min 50% | 10.173 | 0.001 |
| 21 |  | Leaf: Moi_mo_ | -36.911 | plus 10% | 0.577 | 0.448 |
| 22 |  |  |  | min 10% | 0.208 | 0.649 |
| 23 |  |  |  | plus 50% | 3.898 | 0.048 |
| 24 |  |  |  | min 50% | 3.898 | 0.048 |

**Table SVIb: The differences between the standard time budget model’s predictions (Expected N) and the predictions of the alternative versions of the model (Amended N) using one of the 24 alternative time budget equations (see Table S6a) that were created to test the robustness of the equations to small or large amendments of the slope values for different variables.** The number (Test1 to Test24) corresponds to the number given in the first column of Table SVIa. Amended N presents the predictions of the amended model for the arboreal guenons to be Present or Absent, whilst Expected N represents the predictions of the standard model. Please note that these values are independent of actual distribution patterns because that is not the purpose of these Chi-square tests.

|  |  | **Amended N** | **Expected N** | **Residual** |
| --- | --- | --- | --- | --- |
| **Test1** |  |  |  |  |
|  | **Absent** | 69 | 63 | 6 |
|  | **Present** | 133 | 139 | -6 |
| **Test2** |  |  |  |  |
|  | **Absent** | 59 | 63 | -4 |
|  | **Present** | 143 | 139 | 4 |
| **Test_3** |  | |  |  |
|  | **Absent** | 91 | 63 | 28 |
|  | **Present** | 111 | 139 | -28 |
| **Test_4** |  | |  |  |
|  | **Absent** | 39 | 63 | -24 |
|  | **Present** | 163 | 139 | 24 |
| **Test_5** |  | |  |  |
|  | **Absent** | 74 | 63 | 11 |
|  | **Present** | 128 | 139 | -11 |
| **Test_6** |  | |  |  |
|  | **Absent** | 57 | 63 | -6 |
|  | **Present** | 145 | 139 | 6 |
| **Test_7** |  | |  |  |
|  | **Absent** | 100 | 63 | 37 |
|  | **Present** | 102 | 139 | -37 |
| **Test_8** |  | |  |  |
|  | **Absent** | 38 | 63 | -25 |
|  | **Present** | 164 | 139 | 25 |
| **Test_9** |  | |  |  |
|  | **Absent** | 47 | 63 | -16 |
|  | **Present** | 155 | 139 | 16 |
| **Test_10** |  | |  |  |
|  | **Absent** | 82 | 63 | 19 |
|  | **Present** | 120 | 139 | -19 |
| **Test_11** |  | |  |  |
|  | **Absent** | 16 | 63 | -47 |
|  | **Present** | 186 | 139 | 47 |
| **Test_12** |  | |  |  |
|  | **Absent** | 124 | 63 | 61 |
|  | **Present** | 78 | 139 | -61 |
| **Test_13** |  | |  |  |
|  | **Absent** | 58 | 63 | -5 |
|  | **Present** | 144 | 139 | 5 |
| **Test_14** |  | |  |  |
|  | **Absent** | 76 | 63 | 13 |
|  | **Present** | 126 | 139 | -13 |
| **Test_15** |  | |  |  |
|  | **Absent** | 34 | 63 | -29 |
|  | **Present** | 168 | 139 | 29 |
| **Test_16** |  | |  |  |
|  | **Absent** | 108 | 63 | 45 |
|  | **Present** | 94 | 139 | -45 |
| **Test_17** |  | |  |  |
|  | **Absent** | 69 | 63 | 6 |
|  | **Present** | 133 | 139 | -6 |
| **Test_18** |  | |  |  |
|  | **Absent** | 60 | 63 | -3 |
|  | **Present** | 142 | 139 | 3 |
| **Test_19** |  | |  |  |
|  | **Absent** | 89 | 63 | 26 |
|  | **Present** | 113 | 139 | -26 |
| **Test_20** |  | |  |  |
|  | **Absent** | 42 | 63 | -21 |
|  | **Present** | 160 | 139 | 21 |
| **Test_21** |  | |  |  |
|  | **Absent** | 68 | 63 | 5 |
|  | **Present** | 134 | 139 | -5 |
| **Test_22** |  | |  |  |
|  | **Absent** | 60 | 63 | -3 |
|  | **Present** | 142 | 139 | 3 |
| **Test_23** |  | |  |  |
|  | **Absent** | 76 | 63 | 13 |
|  | **Present** | 126 | 139 | -13 |
| **Test_24** |  | |  |  |
|  | **Absent** | 50 | 63 | -13 |
|  | **Present** | 152 | 139 | 13 |

**References for Supplementary material**

**APAAT:**

The Assessment of African Protected Areas A characterisation of biodiversity value, ecosystems and threats to inform the effective allocation of conservation funding A.J. Hartley, A. Nelson, P. Mayaux and J-M. Grégoire

APAAT accessed in 2013 and 2014: http://dopa.jrc.ec.europa.eu/sites/dopa.jrc.ec.europa.eu/files/AssessmentOfAfricanProtectedAreas_EUR22780%20%281%29.pdf

**WCMC:**

UNEP WCMC database of protected areas was accessed between 2003-2006

**APAAT and WCMC have been incorporated into DOPA: Digital Observatory for Protected Areas (http://dopa.jrc.ec.europa.eu/node/4)**

**Other references:**

Aalangdong, O. I. (2009). Rapid Assessment of Large Mammals in the Ajenjua Bepo and Mamang River Forest Reserves in the Abirem North district, eastern region, Ghana. In *RAP Bulletin of Biological Assessment: A Rapid Biodiversity Assessment of the Ajenjua Bepo and Mamang River Forest Reserves, Ghana* (pp. 58–60). Conservation International: BioOne.

Aldrich-Blake, F. P. G. (1970). Problems of social structure in forest monkeys. In J. H. Crook (Ed.), *Social behaviour in birds and mammals* (pp. 79–101). London: Academic Press Inc.

Alonso, L. E., Laugenie, F., & Rondeau, G. (2005). *A rapid biological assessment of two classified forests in south western Côte d’Ivoire*. *RAP bulletin of biological assessment* (Vol. 34). Washington D.C.: Conservation International.

Baranga, D. (2004). Forest fragmentation and primates’ survival status in non‐reserved forests of the ‘Kampala area’, Uganda. *African Journal of Ecology*, *42*(s1), 70–77.

Barrie, A., Zwuen, S., Kota, A. N., Lou Sr, M., & Luke, R. (2007). Rapid survey of large mammals of North Lorma, Gola and Grebo National Forests. In *RAP bulletin of biological assessment: A Rapid Biological Assessment of North Lorma, Gola and Grebo National Forests, Liberia* (Vol. 44, pp. 59–64). Conservation International.

Bassey, E. (2012). *Afi Quarterly report October-December 2012*.

Beeson, M., Tame, S., Keeming, E., & Lea, S. E. G. (1996). Food habits of guenons (Cercopithecus spp.) in Afro-montane forest. *African J Ecol*, *34*(2), 202–210. doi:10.1111/j.1365-2028.1996.020-89020.x

Bermejo, M. (1999). Status and conservation of primates in Odzala National Park, Republic of the Congo. *Oryx*, *33*(4), 323–331.

Blake, S. (1993). *A reconnaissance survey in the Likouala swamps of northern Congo and its implications for conservation*. University of Edinburgh.

Blom, A., Alers, M. P. T., Feistner, A. T. C., Barnes, R. F. W., & Barnes, K. L. (1992). Primates in Gabon--current status and distribution. *Oryx*, *26*(4), 223–234.

Brugiere, D., Gautier, J. P., Moungazi, A., Gautier-hion, A., & Brugière, D. (2002). Primate diet and biomass in relation to vegetation composition and fruiting phenology in a rain forest in Gabon. *International Journal of Primatology*, *23*(5), 999–1024.

Brugière, D., Sakom, D., & Gautier-Hion, A. (1999). *Primates simiens en forêt de Ngotto*.

Brugière, D., Sakom, D., & Sinassonasibe, J. P. (1999). *Estimation des densités et analyse du comportement nidificateur des gorilles et chimpanzés en forêt de Ngotto*.

Butynski, T. M. (1990). Comparative ecology of blue monkeys (Cercopithecus mitis) in high- and low-density subpopulations. *Ecological Monographs*, *60*, 1–26. doi:10.2307/1943024

Butynski, T. M. (2013a). Cercopithecus dryas Dryad monkey (Salongo monkey). In T. M. Butynski, J. Kingdon, & J. Kalina (Eds.), *Mammals of Africa: Volume II Primates* (pp. 307–309). London: Bloomsbury.

Butynski, T. M. (2013b). Allochrocebus preussi: Preuss’s monkey. In T. M. Butynski, J. Kingdon, & J. Kalina (Eds.), *Mammals of Africa: Volume II Primates* (pp. 293–295). London: Bloomsbury.

Butynski, T. M., & Kingdon, J. (2013). Cercopithecus erythrotis: red-eared monkey (red-nosed monkey). In *Mammals of Africa: Volume II Primates* (pp. 373–375). London: Bloomsbury.

Buzzard, P. J. (2003). Ecological partitioning in tai forest guenons: Cercopithecus campbelli, C. petaurista, C. diana. *American Journal of Physical Anthropology*, 73.

Buzzard, P. J. (2004). *Interspecific competition among Cercopithecus campbelli, C. petaurista, and C. diana at Taï Forest, Cote d’Ivoire*. Columbia University, New York.

Bytynski, T. M., & McCullough, J. (2007). *A Rapid Biological Assessment of Lokutu, Democratic Republic of Congo*. *RAP Bulletin of Biological Assessments* (Vol. 46). Conservation International. doi:10.1896/978-1-934151-04-4

Campbell, G., Teichroeb, J., & Paterson, J. D. (2008). Distribution of diurnal primate species in Togo and Bénin. *Folia Primatologica*, *79*(1), 15–30.

Centre d’echange d’information du Benin, C. (2004). *Monopgraphie nationale de la diversite Biologique*.

Chapman, C. A., & Chapman, L. J. (2000). Constraints on group size in red colobus and red-tailed guenons: examining the generality of the ecological constraints model. *International Journal of Primatology*, *21*(4), 565–585.

Chapman, C. A., Chapman, L. J., Cords, M., Gathua, J. M., Gautier-Hion, A., Lambert, J. E., *et al.* (2002). Variation in the diets of Cercopithecus species: Differences within forests, among forests, and across species. In M. E. Glenn & M. Cords (Eds.), *The guenons: diversity and adaptation in African monkeys* (pp. 325–350). New York: Kluwer Acad/Plenum Publ.

Chapman, C. A., Gautier-Hion, A., Oates, J. F., & Onderdonk, D. A. (1999). African primate communities: determinants of structure and threats to survival. In J. J. Fleagle, C. Janson, & K. E. Reed (Eds.), *Primate communities* (pp. 1–37). Cambridge: Cambridge University Press.

Christy, P., Lahm, S. A., Pauwels, O. S. G., & Vande Weghe, J. P. (2008). *Check-list des amphibiens, reptiles, oiseaux et mammiferes des parcs nationaux du Gabon. Checklist of Amphibians, Reptiles, Birds and Mammals of the National Parks of Gabon*. Washington: Smithsonian Institution.

Coad, L. M., Tanga, J.-J., Maisels, F., Abernethy, K., Bout, N., Fay, M., *et al.* (2010). New range limits of the sun-tailed monkey, Cercopithecus solatus, in Central Gabon. *Primate Conservation*, (25), 33–41.

Colyn, M., & Deleporte, P. (2002). Biogeographic analysis of central African forest guenons. In Glenn & M. Cords (Eds.), *The guenons: diversity and adaptation in African monkeys* (pp. 61–78). New York: Kluwer Acad/Plenum Publ.

Convention sur la Diversite Biologique. (n.d.). *Etat de la diversité biologique en République Démocratique du Congo*.

Cords, M. (1986). Interspecific and intraspecific variation in diet of 2 forest guenons, Cercopithecus ascanius and C. mitis. *Journal of Animal Ecology*, *55*(3), 811–827.

Cords, M. (1989). Vigilance and mixed-species association of some East African forest monkeys. *Behavioral Ecology And Sociobiology*, *26*, 297–300.

Cords, M. (2012). The 30-Year Blues: What We Know and Don’t Know About Life History, Group Size, and Group Fission of Blue Monkeys in the Kakamega Forest, Kenya. In P. M. Kappeler & D. P. Watts (Eds.), *Long-Term Field Studies of Primates* (pp. 289–311). Heidelberg: Springer.

Curtin, S. H. (2002). Diet of the Roloway monkey, Cercopithecus diana roloway, in Bia National Park, Ghana. In M. E. Glenn & M. Cords (Eds.), *The guenons: diversity and adaptation in African monkeys* (pp. 351–371). New York: Kluwer Acad/Plenum Publ.

Davenport, T. R. B., Nowak, K., & Perkin, A. (2014). Priority primate areas in Tanzania. *Oryx*, *48*(1), 39–51.

de Jong, Y. A., & Butynski, T. M. (2010). *Assessment of the primates, large mammals and birds of the Mathews Range Forest Reserve, central Kenya*. Washington D.C.

de Vos, A., & Omar, A. (1971). Territories and movements of Sykes monkeys (Cercopithecus mitis kolbi Neuman) in Kenya. *Folia Primatologica*, *16*(3–4), 196–223.

Decker, B. S. (1995). Survey of De Brazza’s monkey (Cercopithecus neglectus Schlegel) in the tororo district of eastern uganda and trans-nzoia and west pokot districts of western kenya. *Journal of East African Natural History*, *84*(1), 25–34.

Dinesen, L., Lehmberg, T., Rahner, Ma. C., & Fjeldsa, J. (2001). Conservation priorities for the forests of the Udzungwa Mountains, Tanzania, based on primates, duikers and birds. *Biological Conservation*, *99*, 223–236.

Eltringham, S. K., Morley, R. J., Kingdon, J., Coe, M. J., & McWilliam, N. C. (1999). Checklist: mammals of Mkomazi. In M. Coe, N. McWilliam, G. Stone, & M. Packer (Eds.), *Mkomazi: the ecology, biodiversity and conservation of a Tanzanian savanna* (pp. 505–510). Royal Geographical Society.

Emerson, S. E., Brown, J. S., & Linden, J. D. (2011). Identifying Sykes’ monkeys’, *Cercopithecus albogularis erythrarchus*, axes of fear through patch use. *Animal Behaviour*, *81*(2), 455–462. doi:DOI: 10.1016/j.anbehav.2010.11.018

Fairgrieve, C., & Muhumuza, G. (2003). Feeding ecology and dietary differences between blue monkey (Cercopithecus mitis stuhlmanni Matschie) groups in logged and unlogged forest, Budongo Forest Reserve, Uganda. *AFRICAN Journal of Ecology*, *41*(2), 141–149.

Fairgrieve, C., & Muhumuza, G. (2003). Feeding ecology and dietary differences between blue monkey (Cercopithecus mitis stuhlmanni Matschie) groups in logged and unlogged forest , Budongo Forest Reserve, Uganda. *African Journal of Ecology*, *41*, 141–149.

Fashing, P. J., & Cords, M. (2000). Diurnal primate densities and biomass in the Kakamega Forest: an evaluation of census methods and a comparison with other forests. *American Journal of Primatology*, *50*(2), 139–152. doi:10.1002/(SICI)1098-2345(200002)50:2<139::AID-AJP4>3.0.CO;2-N

Fischer, F., Gross, M., & Kunz, B. (1999). Primates of the Comoé National Park, Ivory Coast. *African Primates*, *4*(1–2), 10–15.

Fleury, M., & Gautier-Hion, A. (1999). Seminomadic ranging in a population of black colobus (Colobus satanas) in Gabon and its ecological correlates. *International Journal of Primatology*, *20*(4), 491–509.

Galat-Luong, A., Galat, G., Glenn, M. E., & McGraw, W. S. (2013). Cercopithecus lowei: Lowe’s monkey. In T. M. Butynski, J. Kingdon, & J. Kalina (Eds.), *Mammals of Africa: Volume II Primates* (pp. 325–327). London: Bloomsbury.

Galat, G., Galat-Luong, A., Glenn, M. E., & McGraw, W. S. (2013). Cercopithecus campbelli: Campbell’s monkey. In T. M. Butynski, J. Kingdon, & J. Kalina (Eds.), *Mammals of Africa: Volume II Primates* (pp. 328–330). London: Bloomsbury.

Garine-wichatitsky, M. D. E., Cheke, R. A., & Lazaro, D. (2001). Effects of tsetse targets on mammals and birds in Kasungu National Park , Malawi, 869–891.

Gautier-Hion, A. (1980). Seasonal-Variations of Diet Related to Species and Sex in a Community of Cercopithecus Monkeys. *Journal Of Animal Ecology*, *49*(1), 237–269.

Gautier-Hion, A. (2013). Cercopithecus wolfi Wolf’s Monkey. In T. M. Butynski, J. Kingdon, & J. Kalina (Eds.), *Mammals of Africa: Volume II Primates* (pp. 333–335). London: Bloomsbury. doi:10.1007/978-3-642-73769-5_2

Gautier-Hion, A., & Brugière, D. (2005). Significance of Riparian Forests for the Conservation of Central African Primates. *International Journal of Primatology*, *26*(3), 515–523. doi:10.1007/s10764-005-4363-1

Gautier-Hion, A., Colyn, M., & Gautier, J.-P. (1999). *Histoire Naturelle des primates d’Afrique Centrale*. ECOFAC.

Gevaerts, H. (1992). Birth Seasons of Cercopithecus, Cercocebus and Colobus in Zaire. *Folia Primatologica*, *59*(2), 105–113.

Glenn, M. E., Bensen, K. J., & Matsuda Goodwin, R. (2013). Cercopithecus mona: Mona monkey. In T. M. Butynski, J. Kingdon, & J. Kalina (Eds.), *Mammals of Africa: Volume II Primates* (pp. 322–324). London: Bloomsbury.

Glenn, M. E., Matsuda, R., & Bensen, K. J. (2002). Unique behavior of the mona monkeys (Cercopithecus mona): all-male groups and copulation calls. In M. E. Glenn & M. Cords (Eds.), *The guenons: diversity and adaptation in African monkeys* (pp. 133–145). New York: Kluwer Acad/Plenum Publ.

Gonedelé Bi, S., Koné, I., Bitty, A. E., Bene Koffi, J. C., Akpatou, B., & Zinner, D. (2012). Distribution and conservation status of catarrhine primates in Côte d’Ivoire (West Africa). *Folia Primatologica*, *83*(1), 11–23.

Griffin, M., & 1Griffin, M. (1998). The species diversity, distribution and conservation of Namibian mammals. *Biodiversity And Conservation*, *7*(4), 483–494.

Grubb, P., & Powell, C. B. (1999). Discovery of red colobus monkeys (Procolobus badius) in the Niger Delta with the description of a new and geographically isolated subspecies. *Journal Of Zoology*, *248*(1), 67–73. doi:10.1017/S0952836999005075

Hall, J. S., White, L., Williamson, E. A., Inogwabini, B.-I., & Ilambu, O. (2003). Distribution, abundance, and biomass estimates for primates within Kahuzi-Biega lowlands and adjacent forest in Eastern DRC. *African Primates*, *6*(1&2), 35–42.

Hart, J. A., Butynski, T. M., Sarmiento, E. E., & de Jong, Y. A. (2013). Cercopithecus Hamlyni: Owl-faced monkey (Hamlyn’s monkey). In T. M. Butynski, J. Kingdon, & J. Kalina (Eds.), *Mammals of Africa: Volume II Primates* (pp. 339–344). London: Bloomsbury.

Henzi, S. P., & Lawes, M. J. (1987). Breeding season influxes and the behaviour of adult male samango monkeys (Cercopithecus mitis albogularis). *Folia Primatologica*, *48*, 125–136.

Hill, C. M., & Oates, J. F. (2013). Cercopithecus diana Diana monkey. In T. M. Butynski, J. Kingdon, & J. Kalina (Eds.), *Mammals of Africa: Volume II Primates* (pp. 310–312). London: Bloomsbury.

Kadiri, N. G. (2014). *Yankari Quarterly report April-June 2014*.

Kano, T. (1996). Male rank order and copulation rate in a unit-group of bonobos at Wamba, Zaïre. In W. C. McGrew, L. F. Marchant, & T. Nishida (Eds.), *Great ape societies* (pp. 135–145). Cambridge: Cambridge University Press.

Kaplin, B. A. (2001). Ranging behavior of two species of guenons (Cercopithecus lhoesti and C. mitis doggetti) in the Nyungwe Forest Reserve, Rwanda. *International Journal of Primatology,* *22*(4), 521–548.

Kaplin, B. A., & Moermond, T. C. (1998). Variation in seed handling by two species of forest monkeys in Rwanda. *American Journal Of Primatology*, *45*(1), 83–101.

Kaplin, B. A., & Moermond, T. C. (2000). Foraging ecology of the mountain monkey (Cercopithecus l’hoesti): Implications for its evolutionary history and use of disturbed forest. *American Journal Of Primatology*, *50*(4), 227–246.

Karere, G. M., Oguge, N. O., Kirathe, J., Muoria, P. K., Moinde, N. N., & Suleman, M. A. (2004). Population sizes and distribution of primates in the lower Tana River forests, Kenya. *International Journal of Primatology*, *25*(2), 351–365.

King, T. (2008). Detectability and conservation of De Brazza’s Monkey (Cercopithecus neglectus) in the lesio-louna and south-west lefini reserves, bateke plateau, republic of congo. *Primate Conservation*, *23*(1), 39–44.

Kormos, R., Boesch, C., Bakarr, M. I., & Butynski, T. M. (2003). *Status Survey and Conservation Action Plan - West African chimpanzees*.

Lambrechts, C., Woodley, B., Church, C., & Gachanja, M. (2003). *Aerial survey of the destruction of the Aberdare Range forests*. *Division of Early Warning and Assessment, UNEP*.

Lawes, M. J. (1991). Diet of samango monkeys (Cercopithecus mitis erythrarchus) in the Cape Vidal Dune Forest, South-Africa. *Journal of Zoology*, *224*, 149–173.

Lawes, M. J., Henzi, S. P., & Perrin, M. R. (1990). Diet and feeding behavior of Samango monkeys (Cercopithecus mitis labiatus) in Ngoye Forest, South-Africa. *Folia Primatologica*, *54*(1–2), 57–69.

Maisels, F., Pambou Makaya, Q., & Onononga, J.-R. (2007). Confirmation of the Presence of the Red-capped Mangabey (Cercocebus torquatus) in Mayumba National Park, Southern Gabon, and Conkouati-Douli National Park, Southern Republic of Congo. *Primate conservation*, *22*(1), 111–115.

Marshall, A. R., Topp-Jørgensen, J. E., Brink, H., & Fanning, E. (2005). Monkey abundance and social structure in two high-elevation forest reserves in the Udzungwa Mountains of Tanzania. *International Journal of Primatology*, *26*(1), 127–145.

Mate, C., Colell, M., & Escobar, M. (1995). Preliminary observations on the ecology of forest Cercopithecidae in the Lokofe-Ikomaloki region (Ikela, Zaire). *Folia Primatologica*, *64*(4), 196–200.

Mathews, A., & Matthews, A. (2002). Distribution, population density, and status of sympatric cercopithecids in the Campo-Ma’an area, Southwestern Cameroon. *Primates*, *43*(3), 155–168.

Mathot, L., Ikoli, F., & Missilou, B. R. (2006). *Rapport annuel de monitoring de la faune du Projet Lésio-Louna, 2006*.

Matthews, A. (2004). Survey of gorillas (Gorilla gorilla gorilla) and chimpanzees (Pan troglodytes troglodytes) in Southwestern Cameroon. *Primates*, *45*(1), 15–24.

McGraw, W. S. (1994). Census, habitat preference, and polyspecific associations of six monkeys in the lomako forest, Zaire. *American Journal Of Primatology*, *34*, 295–307.

McGraw, W. S. (2005). Update on the Search for Miss Waldron’s Red Colobus Monkey. *International Journal of Primatology*, *26*(3), 605–619. doi:10.1007/s10764-005-4368-9

McGraw, W. S., Zuberbühler, K., & Noë, R. (2007). *Monkeys of the Taï forest: an African primate community* (Vol. Cambridge). Cambridge: Cambridge University Press.

Ministério do urbanismo e ambiente. (2006). *Primeiro Relatório Nacional para a Conferência das Partes da Convenção da Diversidade Biológica*. Luanda, Angola.

Moreno-Black, G., & Maples, W. R. (1977). Differential habitat utilization of four cercopithecidae in a Kenyan forest. *Folia Primatologica*, *27*(2), 85–107.

Morgan, B. J., Wild, C., & Ekobo, A. (2003). Newly discovered gorilla population in the Ebo Forest, Littoral Province, Cameroon. *International Journal of Primatology*, *24*(5), 1129–1137.

Mugambi, K. G., Butynski, T. M., Suleman, M. A., & Ottichilo, W. (1997). The vanishing De Brazza’s monkey (Cercopithecus neglectus Schlegel) in Kenya. *International Journal of Primatology*, *18*(6), 995–1004.

Mwenja, I. (2007). A New Population of De Brazza’s Monkey in Kenya. *Primate Conservation*, *22*(1), 117–122. doi:10.1896/052.022.0111

NBA. (2011). *National biodiversity assessment 2011*.

Nishida, T., & Hosaka, K. (1996). Coalition strategies among adult male chimpanzees of the Mahale Mountains, Tanzania. In W. C. McGrew, L. F. Marchant, & T. Nishida (Eds.), *Great ape societies* (pp. 114–134). Cambridge: Cambridge University Press.

Nzigidahera, B. (2000). *Analyse de la diversite biologique vegetale nationale et identification des priorites pour sa conservation*. Bujumbura.

Oates, J. F. (1977). The guereza and its food. In T. H. Clutton-Brock (Ed.), *Primate ecology: studies of feeding and ranging behaviour in lemurs, monkeys and apes* (pp. 275–321). London: Acadamic Press.

Oates, J. F., Whitesides, G. H., Davies, A. G., Waterman, P. G., Green, S. M., Dasilva, G. L., & Mole, S. (1990). Determinants of variation in tropical forest primate biomass: new evidence from West Africa. *Ecology*, *71*(1), 328–343.

Owiunji, I., Nkuutu, D., Kujirakwinja, D., Liengola, I., Plumptre, A. J., Nsanzurwimo, A., *et al.* (2005). The biodiversity of the Virunga Volcanoes. *Unpublished report, Wildlife Conservation Society, New York*.

Payne, H. F. P., Lawes, M. J., & Henzi, S. P. (2003). Competition and the exchange of grooming among female samango monkeys (Cercopithecus mitis erythrarchus). *Behaviour*, *140*, 453–471.

Rowcliffe, J. M., de Merode, E., & Cowlishaw, G. (2004). Do wildlife laws work? Species protection and the application of a prey choice model to poaching decisions. *Proceedings of the Royal Society of London B: Biological Sciences*, *271*(1557), 2631–2636.

Sarmiento, E. E. (2013). Allochrocebus lhoesti: L’Hoest’s monkey. In T. M. Butynski, J. Kingdon, & J. Kalina (Eds.), *Mammals of Africa: Volume II Primates* (pp. 296–300). London: Bloomsbury.

Schlichte, H.-J. (1978). The ecology of two groups of blue monkeys, Cercopithecus mitis stuhlmanni, in an isolated habitat of poor vegetation. In G. G. Montgomery (Ed.), *The ecology of arboreal folivores* (pp. 505–517). Washington DC: Smithsonian Institution Press.

Sery, G. B., Zinner, D., Koné, I., Bi, Z. G., Akpatou, B., Koffi Bené, J. C., *et al.* (2006). A West African black-and-white colobus monkey, Colobus polykomos dollmani Schwarz, 1927, facing extinction. *Primate Conservation*, *21*, 55–61.

Struhsaker, T. T. (1969). Correlates of ecology and social organization among African cercopithecines. *Folia Primatologica*, *11*(1–2), 80–118.

Struhsaker, T. T., & Leland, L. (1979). Socioecology of five sympatric monkey species in the Kibale Forest, Uganda. *Advances in the study of Behavior*, *9*, 159–228.

Tanzania National Parks. (1979). *Lake Manyara National Park: A field guide*. Arusha: Tanzania Litho Ltd.

Tashiro, Y. (2006). Frequent insectivory by two guenons (Cercopithecus lhoesti and Cercopithecus mitis) in the Kalinzu Forest, Uganda. *Primates*, *47*, 170–173. doi:10.1007/s10329-005-0160-x

Teichroeb, J. A., Saj, T. L., Paterson, J. D., & Sicotte, P. (2003). Effect of group size on activity budgets of Colobus vellerosus in Ghana. *International Journal of Primatology*, *24*(4), 743–758.

Thomas, S. C. (1991). Population-Densities and Patterns of Habitat Use among Anthropoid Primates of the Ituri Forest, Zaire. *BIOTROPICA*, *23*(1), 68–83.

Thompson, J. A. M. (1997). *The History, Taxonomy and Ecology of the Bonobo (Pan Paniscus Schwarz, 1929) with a First Description of a Wild Population Living in a Forest/savanna Mosaic Habitat*. University of Oxford.

Tolo, C. U., Baranga, J., & Kagoro-Rugunda, G. (2008). Dietary selection of L’Hoest monkeys in Kalinzu forest reserve, southwestern Uganda. *African Journal of Ecology*, *46*(2), 149–157. doi:10.1111/j.1365-2028.2007.00826.x

Tutin, C. E. G. (1999). Fragmented living: Behavioural ecology of primates in a forest fragment in the Lope Reserve, Gabon. *Primates*, *40*(1), 249–265.

Tutin, C. E. G., Ham, R. M., White, L. J. T., & Harrison, M. J. S. (1997). The primate community of the Lopé Reserve, Gabon: diets, responses to fruit scarcity, and effects on biomass. *American journal of primatology*, *42*(1), 1–24. doi:10.1002/(SICI)1098-2345(1997)42:1<1::AID-AJP1>3.0.CO;2-0

Tweheyo, M., & Obua, J. (2001). Feeding habits of chimpanzees (Pan troglodytes), red-tail monkeys (Cercopithecus ascanius schmidti) and blue monkeys (Cercopithecus mitis stuhlmanii) on figs in Budongo Forest Reserve, Uganda. *African Journal of Ecology*, *39*(2), 133–139.

Uehara, S. (2003). Population densities of diurnal mammals sympatric with the chimpanzees of the Mahale Mountains, Tanzania: comparison between census data of 1996 and 2000. *African Study Monographs*, *24*(3), 169–179.

van der Zee, D., & Skinner, J. D. (1977). Preliminary observations on samango and vervet monkeys near Lake Sibayi. *South African Journal of Science*, *73*, 381–382.

Waltert, M., Lien, Faber, K., & Muhlenberg, M. (2002). Further declines of threatened primates in the Korup Project Area, south-west Cameroon. *Oryx*, *36*(3), 257–265.

Werre, J. L. R. (2000). *Ecology and behavior of the Niger Delta red colobus (Procolobus badius epieni)*. The City University of New York, New York.

White, L. J. T. (1994). Biomass of Rain-Forest Mammals in the Lope Reserve, Gabon. *Journal Of Animal Ecology*, *63*(3), 499–512.

Whitesides, G. H. (1989). Interspecific associations of Diana monkeys, Cercopithecus diana , in Sierra Leone, West Africa: Biological significance or chance? *Animal Behaviour*, *37*(5), 760–776.

Williams, J. G. (1967). *A field guide to the national parks of East Africa* (2nd ed., Vol. 1). Glasgow: William Collins Sons and Co Ltd.

Wolfheim, J. H. (1983). *Primates of the world: distribution, abundance and conservation*. Seatle: University of Washington Press.
